# Supplementary material for: Paternity success for resident and non-resident males and their influences on paternal sibling cohorts in Japanese macaques (Macaca fuscata) on Shodoshima Island
Source: PLoS One. 2024 Sep 19;19(9):e0309056. doi: 10.1371/journal.pone.0309056 (PMC11412542; doi:10.1371/journal.pone.0309056)
Supplement: S1 Table — (DOCX) [file pone.0309056.s001.docx]

**S1 Table. Information for candidate fathers.**

| ID | 2017 | 2018 | | 2019 | | 2020 | | 2021 | | 2022 |
| --- | --- | --- | --- | --- | --- | --- | --- | --- | --- | --- |
|  | m | nm | m | nm | m | nm | m | nm | m | nm |
| SN | D/A | D/A | D/A | D/A | D/A | D/A | D/A | D/A | *absent* | *absent* |
| TY | NR/A* | D/A | D/A | D/A | D/A | D/A | D/A | D/A | D/A | D/A |
| YG | S/SA | S/A | S/A | S/A | S/A | S/A | S/A | S/A | D/A | D/A |
| YW | S/A | S/A | S/A | S/A | S/A | S/A | *absent* | *absent* | *absent* | *absent* |
| ClS | S/SA | S/SA | S/SA | S/SA | S/SA | S/A | S/A | *absent* | *absent* | *absent* |
| TR | D/A | *absent* | *absent* | *absent* | *absent* | *absent* | *absent* | *absent* | *absent* | *absent* |
| SB | D/A | *absent* | *absent* | *absent* | *absent* | *absent* | *absent* | *absent* | *absent* | *absent* |
| TmS | *immature* | S/SA | S/SA | S/SA | S/SA | S/SA | S/SA | S/A | S/A | S/A |
| BkS | *immature* | S/SA | S/SA | S/SA | S/SA | S/SA | S/SA | S/A | S/A | S/A |
| WH | N/A | N/A | N/A | S/SA | S/SA | S/SA | S/SA | S/A | S/A | S/A |
| NP | N/A | N/A | NR/A | *absent* | NR/A | *absent* | NR/A | *absent* | NR/A | *absent* |
| TN | N/A | N/A | N/A | *absent* | NR/A | *absent* | NR/A | *absent* | NR/A | *absent* |
| BrS | *immature* | *immature* | *immature* | *immature* | *immature* | S/SA | D/SA | D/SA | D/SA | D/SA |
| BL | N/A | N/A | N/A | S/SA | S/SA | S/SA | S/SA | S/SA | S/SA | S/SA |
| KR | *immature* | *immature* | *immature* | S/SA | S/SA | S/SA | S/SA | S/SA | S/SA | S/SA |
| RZ | N/A | N/A | N/A | S/SA | S/SA | S/SA | S/SA | S/SA | S/SA | S/SA |
| HP | N/A | N/A | N/A | S/SA | S/SA | S/SA | S/SA | *absent* | *absent* | *absent* |
| NmS | *immature* | *immature* | *immature* | *immature* | *immature* | S/SA | S/SA | S/SA | S/SA | S/SA |
| HsS | *immature* | *immature* | *immature* | *immature* | *immature* | *immature* | *immature* | S/SA | S/SA | S/SA |

D, S, and NR indicate dominant, subordinate, and non-resident males, respectively. A and SA indicate adult and subadult males, respectively.

“m” and “nm” indicate mating seasons and non-mating seasons, respectively. N/A represents data that was not available.

Shadow indicates the candidate fathers.

* Although the demographic data for TY was not collected in non-mating season 2017, we classified the male as a non-resident male because he was begun to be observed in the end of September and was not habituated to human.
