# Supplementary material for: Paternity success for resident and non-resident males and their influences on paternal sibling cohorts in Japanese macaques (Macaca fuscata) on Shodoshima Island
Source: PLoS One. 2024 Sep 19;19(9):e0309056. doi: 10.1371/journal.pone.0309056 (PMC11412542; doi:10.1371/journal.pone.0309056)
Supplement: S3 Table — (DOCX) [file pone.0309056.s003.docx]

**S3 Table. Summary for population genetic parameters.**

| Locus | N | No. of alleles | Observed heterozygosity | Expected heterozygosity | Allelic dropout rate |
| --- | --- | --- | --- | --- | --- |
| MFGT22 | 88 | 8 | 0.716 | 0.787 | 0% |
| D5s820 | 88 | 6 | 0.670 | 0.683 | 0% |
| MFGT18 | 88 | 6 | 0.489 | 0.456 | 0% |
| D6s501 | 88 | 8 | 0.875 | 0.826 | 0% |
| D3s1768 | 88 | 5 | 0.648 | 0.683 | 1% |
| D17s1290 | 88 | 8 | 0.693 | 0.712 | 1% |
| MFGT21 | 88 | 7 | 0.261 | 0.335 | 3% |
| MFGT5 | 88 | 3 | 0.443 | 0.471 | 1% |
| D6s493 | 88 | 7 | 0.716 | 0.713 | 1% |
| D14s306 | 88 | 5 | 0.670 | 0.712 | 0% |
| D19s582 | 88 | 6 | 0.693 | 0.667 | 1% |
| D20s484 | 88 | 7 | 0.625 | 0.778 | 4% |
| MFGT27 | 88 | 6 | 0.670 | 0.729 | 0% |
| D7s821 | 88 | 7 | 0.693 | 0.701 | 0% |
| MFGT24 | 88 | 7 | 0.795 | 0.751 | 1% |
| D1s548 | 88 | 4 | 0.511 | 0.478 | 0% |
