# Supplementary material for: Paternity success for resident and non-resident males and their influences on paternal sibling cohorts in Japanese macaques (Macaca fuscata) on Shodoshima Island
Source: PLoS One. 2024 Sep 19;19(9):e0309056. doi: 10.1371/journal.pone.0309056 (PMC11412542; doi:10.1371/journal.pone.0309056)
Supplement: S4 Table — (DOCX) [file pone.0309056.s004.docx]

**S4 Table. Results for paternity of offspring in the B-group.**

| Offspring | Year of birth | Mother | Assigned father | Delta score | Confidence level |
| --- | --- | --- | --- | --- | --- |
| Km18 | 2018 | Km | - | - | - |
| Bs18 |  | Bs | - | - | - |
| Sf18 |  | Sf | - | - | - |
| Ma18 |  | Ma | TY | 14.48 | > 95% |
| Dk18 |  | Dk | YW | 6.00 | > 95% |
| Po18 |  | Po | - | - | - |
| Bv18* |  | Bv | - | - | - |
| Bb18* |  | Bb | SN | 3.88 | > 95% |
| Bs19 | 2019 | Bs | - | - | - |
| Vg19 |  | Vg | - | - | - |
| Ok19 |  | Ok | - | - | - |
| Bk19 |  | Bk | SN | 8.05 | > 95% |
| Rw19 |  | Rw | - | - | - |
| Pe19 |  | Pe | SN | 10.03 | > 95% |
| Sk19 |  | Sk | - | - | - |
| Bb19* |  | Bb | SN | 3.85 | > 95% |
| Tr19* |  | Tr | NP | 10.42 | > 95% |
| Br20 | 2020 | Br | NP | 14.00 | > 95% |
| Km20 |  | Km | - | - | - |
| Am20 |  | Am | YG | 14.27 | > 95% |
| Rs20 |  | Rs | YW | 9.76 | > 95% |
| Bs20 |  | Bs | SN | 10.78 | > 95% |
| Nm20 |  | Nm | NP | 18.58 | > 95% |
| Hk20 |  | Hk | YW | 9.54 | > 95% |
| Rw20 |  | Rw | NP | 16.04 | > 95% |
| Br21 | 2021 | Br | YG | 12.58 | > 95% |
| Rs21 |  | Rs | NP | 20.35 | > 95% |
| Mr21 |  | Mr | YG | 11.43 | > 95% |
| Qp21 |  | Qp | NP | 16.93 | > 95% |
| Qb21 |  | Qb | NP | 19.50 | > 95% |
| Tg21 |  | Tg | YG | 12.79 | > 95% |
| Bs21 |  | Bs | TY | 13.15 | > 95% |
| Vg21 |  | Vg | NP | 19.14 | > 95% |
| Sf21 |  | Sf | YG | 7.73 | > 95% |
| Ok21 |  | Ok | - | - | - |
| Br22 | 2022 | Br | WH | 13.89 | > 95% |
| Km22 |  | Km | WH | 13.00 | > 95% |
| Am22 |  | Am | WH | 16.73 | > 95% |
| Qp22 |  | Qp | TY | 13.02 | > 95% |
| Qb22 |  | Qb | WH | 10.84 | > 95% |
| Bs22 |  | Bs | BrS | 8.99 | > 95% |
| Sf22 |  | Sf | BrS | 5.60 | > 95% |
| Nm22 |  | Nm | NP | 16.95 | > 95% |
| Hk22 |  | Hk | BrS | 8.21 | > 95% |
| Mm22 |  | Mm | YG | 7.86 | > 95% |
| Mt22 |  | Mt | TmS | 7.88 | > 95% |

* Samples for mothers of the individuals were not collected, so paternity analysis was performed under the condition where genotypes for their mothers were unavailable.
