# Supplementary material for: Paternity success for resident and non-resident males and their influences on paternal sibling cohorts in Japanese macaques (Macaca fuscata) on Shodoshima Island
Source: PLoS One. 2024 Sep 19;19(9):e0309056. doi: 10.1371/journal.pone.0309056 (PMC11412542; doi:10.1371/journal.pone.0309056)
Supplement: Attachment — Submitted filename: Response letter.docx [file pone.0309056.s006.docx]

**Response letter**

Reviewer #1: The study “Paternity success for resident and non-resident males and their influences on paternal sibling cohorts in Japanese macaques on Shodoshima Island”, by Ishizuka and collaborators is an interesting report of the reproductive dynamics in Macaca fuscata in Choshikei Monkey Park. The authors identify that a single non-resident male sired almost a third of the individuals born between 2020 and 2022, contradicting what would be expected from social species. This is a very interesting finding, and the manuscript would benefit from the authors bringing the discussion one step further. My comments are written bellow:

> We sincerely thank your review and helpful comments on our manuscript. Based on your comments, we have revised our manuscript significantly. We have responded to each of your comments in detail as follows.

In the “Study subjects” section of the Material and Methods, the authors state that the macaques are “provisioned by park staff (…) and by tourists visiting the park”. Could the feeding have decreased the time the resident macaques spend foraging and permitted them to invest higher energy in reproduction? Also, I would imagine that a higher number of well-fed adult females would mean a higher number of estrous females, lowering the competition pressure among resident and non-resident males. Do the authors think that could interfere with the results obtained?

> As you indicated, the effects of provisioning on their reproduction are possible. So it is important to be careful of interrupting the results.

Many previous studies have been conducted from provisioned populations. Our results were basically consistent with previous results regarding Japanese macaques. Therefore, we considered that our results reflect the general patterns of this species. We thus considered that our data can contribute to better understanding the primate reproductive ecology.

Similarly, the ratio of female to male changed through the years analyzed. How would this impact the results?

> That also can impact the results. Therefore, we included the number of capable females as an explanatory variable in GLMMs. Such effect on paternity success was not significant.

I understand that EGP is calculated through the number of offspring that is sired from non-resident and resident males. But that does not translate directly to reproductive success, which would be calculated using the number of mating attempts by one group of individuals. A better proxy for success would be the number of offspring sired divided by the total number of individuals of each group (residents and non-residents). If there is a higher number of approaching non-resident macaques, them siring 50% of the offspring would mean they have a lower success rate than the resident ones. How many non-resident males were identified, even if they were not sampled?

> The idea that divides the number of sires by the total number of resident or non-resident males may be a good way. However, the total number of non-resident males remains unclear even though most of them were identified. Therefore, we considered that assessing the number of sires for each male (including both resident and non-resident males) should be reasonable.

We identified four non-resident males. We successfully collected samples for three of the four males. We have explained these as below:

L160-161: “We identified four non-resident males and three of them were successfully sampled.”

I feel some information of the group dynamics of Japanese macaques would be useful for the reader to better interpret the results. Do young males usually disperse or stay within the mother’s group after reaching maturity? How related are the adult macaques in the analyzed group? How many years does resident males usually stay in a group and how long it takes for non-resident male to become resident? Does the group change composition usually change between mating and non-mating season? These are some interesting points for the authors to discuss regarding the data obtained.

> As you suggested, we added basic information of Japanese macaques, including group composition, males’ dispersal and tenure, and dominance style. Relatedness among adults was not analyzed in this study. The group composition usually does not change between mating and non-mating seasons. Several non-resident males sporadically appear around the group during mating seasons, and some of them continue to stay in the group after the mating season ends. In such cases, those males are interrupted to immigrate into the group.

We have explained these and revised as follows:

L104-110: “They form multi-male and multi-female bisexual groups, in which females stay philopatric and form strong affiliative relationships with matrilineal female kin [44–51]. Males typically emigrate from their natal group when they are approximately four or five years old [52], and continue to transfer between groups throughout life [53]. The tenure of males in a group is approximately three years [54]. Dominance rank is basically linear and stable in both sexes [54–56], although troop takeovers by non-troop males were reported from the Yakushima population [57].”

The results imply that, in the short term, being a non-resident male would be more advantageous because of the higher reproductive success. Still, group behavior is maintained throughout the Primates clade, including Macaca. Is there a higher survival rate of resident members when compared to non-residents? A longer lifespan may compensate the short-term lower success rate by having a higher total number of offspring and by avoiding putting all reproductive effort on harsh seasons.

> What you indicated is important. However, it is extremely hard to monitor the life history of non-resident males in the wild. We have not found any data that compared the survival rate between resident and non-resident males.

I recommend the authors add to the supplement materials the individual genotypes for the 16 microsatellites analyzed.

> As you suggested, we added the genotype data in Table S2.

Small comments:

• In the abstract, line 41: The phrase “neither higher nor lower” should be changed to “similar”.

> We revised as you suggested.

• In line 67, the sentence “Therefore, the exact number of offspring sired by each non-resident male has been rarely investigated.” is redundant when considering the previous one and should be removed. Same thing happens to the sentence started with “Therefore…” in line 90.

> We deleted these sentences to avoid the redundancy.

• In line 143, change “although all non-resident males were not sampled” to “although most of the non-resident males were not sampled”.

> We added the information about the number of identified non-resident males here. We have revised this part as follows:

L160-161: “We identified four non-resident males and three of them were successfully sampled.”

Reviewer #2: OVERALL COMMENTS:

I am very excited about the topic and data presented in this study. With some careful revision and a clear theoretical structure, this paper will make a valuable contribution to the literature. My specific comments below are sincerely meant to be constructive and encouraging, though I recognize they are extensive. I wish the authors the utmost best in reconsidering their approach and framework for communicating this study.

> We sincerely thank your review and helpful comments on our manuscript. Based on your comments, we have revised our manuscript significantly. We have responded to each of your comments in detail as follows.

Overall, I urge the authors to:

1. Reframe their study with a detailed, species-specific introduction and a clear theoretical approach (e.g. benefits of dominance, alternative mating strategies, female choice, etc.).

> As you suggested, we have added the detail information about Japanese macaques (L102-122). We have also explained the effects of dominance hierarchies and female choice on male reproductive success (L55-64). Please review the revised Introduction.

2. Carefully proof-read the manuscript. There are a number of grammatical errors or careless errors like tables and text disagreeing with each other.

> We have checked the grammatical or careless errors. We have also outsourced the English correction service as the second round. We apologize for the mistakes.

3. Clarify the methodology.

> We have added more explanation about the monitoring of identified individuals (L135-139), and statistical methodology (L215-218).

ABSTRACT:

L29-30 I don’t think you need to broadly discuss mammals. I would focus on primates instead.

> As you suggested, we have specifically focused on primates throughout the manuscript.

L32 I would change “males” to “individuals” to avoid over-using the word “male” or “males”

> We have used “individuals” as you suggested (L34).

L29-34 I was surprised, given the topic, to see no mention of the role of dominance or alternative mating strategies. See my comments on the introduction for more detail.

> As you suggested, we have explained the effects of dominance and female mate choice on male reproductive success in the Abstract. We have revised as follows:

L31-33: “As resident males achieve different degree of reproductive success with regard to various factors, such as male dominance relationships or female mate choice, the degree of reproductive success for non-resident males may vary between individuals.”

L34-38 You say that you examined paternity for 46 individuals, but later say you could only successfully assign paternity for 34 offspring. So didn’t you really examine paternity for 34 individuals?

> As you suggested, we have revised to state that we examined paternity of 34 offspring (L40).

L38 You assessed paternity success, but you also compared it between individuals – correct? I would highlight this.

> Yes, we compared paternity success between males.

L39-40 “We investigated the percentages of paternal sibling dyads…” I don’t understand what this sentence is trying to say.

> To clarify the meaning, we have revised this part as follows:

L42-43: “We quantitatively assessed the percentages of paternal siblings in the same age cohorts”

L41 “Non-resident males sired neither higher nor lower percentage of offspring” – so doesn’t this mean that they sired an equal percentage of offspring?

> We have revised to state “similar” percentage (L43).

L42 Was it the same resident male who was successful both years? Or two different resident males?

> it was the same NON-resident male (The ID was “NP”).

L44-45 This point about female preference seems to come from nowhere and it isn’t clear how or why this would explain your results especially since you haven’t discussed female choice or mating strategies at all.

> Since we do not have quantitative data on female choice, we stopped discussing the effects of female choice in the Abstract. However, we have discussed the effects of female choice on male reproductive success in the Introduction and Discussion.

INTRODUCTION

1. You use the word “rarely” quite a bit in your manuscript. I would remove some of these or exchange for different words.

> We have changed “rarely” to different phrases. In the revised manuscript, the “rarely” has been used only once (L30).

2. You spend a lot of time talking about group-living mammals. The phenomenon you are studying is common enough among primate species that I think you should dive right into primate-specific literature.

> As you suggested, we have specifically focused on “primates” throughout the manuscript.

3. Speaking of primate-specific literature, your introduction is incredibly broad and surface-level. You provide very limited specific theoretical framework, concrete examples, references to previous studies, etc. Overall, this needs significantly more depth.

> We have added more explanation about the effects of dominance ranks (L56-60) and female mate choice (L60-64) on male reproductive success.

4. Japanese macaques are you focal species, but the only information you provide is “They form female-philopatric and multimale/multifemale groups”. This is not nearly enough species-specific information.

> We have added more information about Japanese macaques, such as, group composition, female nepotisms, male dispersal, dominance style, breeding seasonality, and female mate choice. We have revised as follows:

L102-122: “To investigate the paternity success for non-resident males and their contributions to the production of kin-dyads within groups, the Japanese macaque *Macaca fuscata* is an interesting species. They form multi-male and multi-female bisexual groups, in which females stay philopatric and form strong affiliative relationships with matrilineal female kin [44–51]. Males typically emigrate from their natal group when they are approximately four or five years old [52], and continue to transfer between groups throughout life [53]. The tenure of males in a group is approximately three years [54]. Dominance rank is basically linear and stable in both sexes [54–56], although troop takeovers by non-troop males were reported from the Yakushima population [57]. Noteworthily, non-resident males often breed with females of the group, as the percentages of EGP are relatively high among primates [28,31]. This is partially because they have breeding seasonality [58,59], and novel and unfamiliar males attain high reproductive success because they are strongly selected by females for mating partners when females are likely to conceive [19,21,60,61]. Further, the exact number of siring for each non-resident male in the field can also be assessed. During the mating season, several non-resident males sporadically appear around the group and copulate with the group’s females [24,62]. Considering that previous field research succeeded in collecting behavioral data and genetic samples from non-resident males [19,24,63], it is possible to expand on it further to examine the paternity success for non-resident males at the individual level. Moreover, the percentage of paternity by the highest-ranking male is usually low [19,24,64], although one study reported a relatively high percentage [65]. This suggests that the percentages of paternal sibling dyads produced by non-resident males can be assessed quantitatively.”

5. Please revise your introduction to be more concise. The second paragraph particularly is rather repetitive.

> We have revised the Introduction significantly. To avoid the redundancy of the paragraph, we have deleted several unnecessary words or sentences. The total number of words in the paragraph has been changed from 305 to 219.

6. I highly recommend that the authors consider a new framework for this paper that focuses on dominance and alternative mating strategies. This would give you a clearer platform from which to launch your investigation of paternity success and the prevalence of EGP. There are some really great articles on this topic in primates and Japanese macaques particularly. I would consider adding any of the following:

a. Bernstein, I. (1976). Dominance, aggression and reproduction in primate societies. Journal of Theoretical Biology, 60(2), 459–472.

b. Bernstein, I. (1978). Primate dominance hierarchies. La Recherche, 9(91), 663–668

c. Chapais, B. (1983). Reproductive activity in relation to male dominance and the likelihood of ovulation in rhesus monkeys. Behavioral Ecology and Sociobiology, 12, 215–228

d. de Ruiter, J. (1993). Male dominance rank and reproductive success in primate groups. Primates, 34(4), 513–523.

e. Eaton, G. G. (1974). Male dominance and aggression in Japanese macaque reproduction. Advances in Behavioral Biology, 11, 287–297. https:// doi.org/10.1007/978-1-4684-3069-1_13.

f. Eaton, G. G. (1976). The social order of Japanese macaques. Scientific American, 235(4), 96–107.

g. Furuichi, T. (1983). Interindividual distance and influence of dominance on feeding in a natural Japanese macaque troop. Primates, 24(4), 445–455

h. Hayawaka, S., & Soltis, J. (2011). Troop takeover and reproductive success of wild male Japanese macaques on Yakushima Island (Macaca fuscata yakui). International Journal of Zoology, 2011, 1–8.

i. Kutsukake, N., & Nunn, C. L. (2006). Comparative tests of reproductive skew in male primates: The roles of demographic factors and incomplete control. Behavioral Ecology and Sociobiology, 60(5), 695–706. https://doi.org/10.1007/s00265-006-0213-1

j. Kutsukake, N., & Nunn, C. L. (2006). Comparative tests of reproductive skew in male primates: The roles of demographic factors and incomplete control. Behavioral Ecology and Sociobiology, 60(5), 695–706. https://doi.org/10.1007/s00265-006-0213-1

k. Perloe, S. I. (1992). Male mating competition, female choice and dominance in a free‐ranging group of Japanese macaques. Primates, 33(3), 289–304

l. Takahata, Y., Huffman, M. A., Suzuki, S., Koyama, N., Yamagiwa, J., & Yamigawa, J. (1999). Why dominants do not consistently attain high mating and reproductive success: A review of longitudinal Japanese macaque studies. Primates, 40, 143–158. https://doi.org/10.1007/ BF02557707

> Thank you for sharing many important previous researches. We have cited most of these studies (Berstein 1976; De Ruiter 1993; Eaton 1976; Furuichi 1983; Hayakawa & Soltis 2011; Kutsukake & Nunn 2009; Perloe 1992; Takahata et al. 1999) and revised our manuscript significantly.

METHODS

1. It feels like there is a lot of information missing here.

> We have added more information and revised the Methods significantly.

2. L124 says that the table is for 2019-2020, but the table title says 2019-2021. This is an example of the much-needed proof-reading.

> We apologize for the careless error. We have revised here.

3. Please describe the “daily monitoring” in depth

> We have explained the “daily monitoring” in detail as follow:

L135-139: “In April 2018, we started monitoring the presence of identified individuals in the monkey park on a daily basis (five or six days per week), although their presence has not been quantitatively recorded. In February 2019, we successfully completed individual identification of the group, and started systematically recording the daily presence of the members of the group.”

3. L128-129 casually mentions capturing of individuals for wildlife management? This needs to be explained in detail since it could be a significant limitation or influencing element of your study

> From 2019 to 2020, many monkeys disappeared one by one. At the time, we did never observed corpses of the monkeys, so did not know reasons for the disappearance. After a couple of years, we found the governmental documents reporting that the monkeys were captured during the period in the town that includes our study site. Therefore, we suspect that the disappearance was due to the captures by the government. However, there is no evidence confirming that the animals were actually captured by the government.

As you pointed out, the effect of the disappearance on the results can be possible. However, it still remains unclear whether the disappearance was a natural catastrophic event, or was unnaturally caused by humans. Therefore, we considered that describing just the fact of disappearance and its possible reasons should be appropriate in the manuscript.

4. Looking at Table 1, only 41/81 group members are accounted for. Are the other 41 juveniles and infants? Please expand this table.

> Yes, we have indicated them in Table 1.

5. Three non-resident males were identified, but how many resident males? Of those, how many were dominant or subordinate? We need a lot more details here.

> We have clarified the number of resident males, including dominant and subordinate males, between 2019 and 2021 as follows:

L171-172: “Two to three dominant, eight to ten subordinate, and two identified non-resident males were present between 2019 and 2021, respectively.”

6. Japanese macaques form despotic linear hierarchies. Why was no effort made to look at the hierarchy using a David’s Score or an Elo Rating or some other measure?

> As you pointed out, we agree the importance of dominance hierarchies in this study. However, it was difficult to collect the dominance-related behaviors of males throughout the five years.

The observation of the study subjects was mainly conducted by YK, who is a park staff of the monkey park and is coauthored in this submission. She could not collect systematic behavioral data, such as antagonistic interactions or signals of submission, because of her own jobs as the park staff. Therefore, there is no data to construct the dominance hierarchies based on the David’s score or Elo rating.

8. Your GLMM – was this using a binomial distribution? What was your link function?

Overall, this needs to be substantially expanded with a lot more specific information about your processes.

> We have explained the error distribution and link function. To clarify the processes of statistical analyses, we have added more explanation about the response variables, and model selection. We have revised as follows:

L213-218: “To analyze the likelihood of siring by each male, we constructed generalized linear mixed models (GLMMs) with binomial distribution and logit link function. We included the percentage of sires for each male in each year as a response variable. Since the total number of sampled males was 10, 10, 12, 12, and 13 in 2017, 2018, 2019, 2020, and 2021, respectively, the total number of data points was 57. The response variable ranged from 0 to 0.40, as the most successful sire’s share in a year was 40% (see the Results).”

I don’t feel I can assess the RESULTS and DISCUSSION based on my concerns and confusion regarding the INTRODUCTION and the METHODS.

> We have revised the Introduction and Methods significantly. We hope that you can assess the Results and Discussion of this study. We are glad if you provide comments on our manuscript.
