# Supplementary material for: Paternity success for resident and non-resident males and their influences on paternal sibling cohorts in Japanese macaques (Macaca fuscata) on Shodoshima Island
Source: PLoS One. 2024 Sep 19;19(9):e0309056. doi: 10.1371/journal.pone.0309056 (PMC11412542; doi:10.1371/journal.pone.0309056)
Supplement: Attachment — Submitted filename: PONE-D-24-26360_reviewer.docx [file pone.0309056.s007.docx]

PLOS ONE

Paternity success for resident and non-resident males and their influences on paternal sibling cohorts in Japanese macaques on Shodoshima Island

--Manuscript Draft--

| Manuscript Number: | PONE-D-24-26360 |
| --- | --- |
| Article Type: | Research Article |
| Full Title: | Paternity success for resident and non-resident males and their influences on paternal sibling cohorts in Japanese macaques on Shodoshima Island |
| Short Title: | Paternity for male Japanese macaques on Shodoshima Island |
| Corresponding Author: | Shintaro ISHIZUKA  Fukuyama University Faculty of Life Science and Biotechnology Graduate School of Biotechnology: Fukuyama Daigaku Seimei Kogakubu Daigakuin Kogaku Kenkyuka Seimei Kogakukei  Fukuyama, JAPAN |
| Keywords: | Japanese macaque, paternity, reproductive skew, extra-group paternity, paternal sibling |
| Abstract: | Reproductive success can be attributed to both resident and non-resident males in non-human primates. However, reproductive success of non-resident males has rarely been investigated at an individual level. As resident males achieve different degree of reproductive success with regard to various factors, such as male dominance relationships or female mate choice, the degree of reproductive success for non- resident males may vary between individuals. As male reproductive success is highly skewed towards specific individuals, the percentage of similar-aged paternal siblings within groups is expected to increase. However, the extent to which each male contributes to the production of cohorts of paternal siblings remains unclear. Here we examined the paternity of offspring born over five consecutive years in a free-ranging group of Japanese macaques Macaca fuscata on Shodoshima Island, Kagawa Prefecture. Genotypes of 87 individuals at 16 autosomal microsatellite loci were analyzed and paternity of 34 offspring was successfully assigned to a single candidate father. We quantitatively assessed paternity success for resident and a few non- resident males whose genetic samples were successfully collected. We quantitatively assessed the percentages of paternal siblings in the same age cohorts produced by those males. Non-resident males sired similar percentage of offspring compared to resident males. A large prime-aged non-resident male was the most successful sire among males in two of the five years. These results provide new insights that male reproductive success could be highly skewed toward a specific non-resident male.  Subadult males had a lower percentage of paternity success, which may be because females may prefer physically mature males. Various males, including non-resident males, contributed to the creation of paternal sibling in the same age cohort. The overall results highlighted that not only resident but also non-resident males play an important role in shaping within-group kin structures. |
| Order of Authors: | Shintaro ISHIZUKA |
|  | Eiji Inoue |
|  | Yuki Kaji |
| Additional Information: |  |
| Question | Response |
| Financial Disclosure  Enter a financial disclosure statement that describes the sources of funding for the work included in this submission. Review the [submission guidelines](http://journals.plos.org/plosone/s/submission-guidelines#loc-financial-disclosure-statement) for detailed | Yes |

| requirements. View published research articles from [*PLOS ONE*](https://journals.plos.org/plosone/) for specific examples.  This statement is required for submission and will appear in the published article if the submission is accepted. Please make sure it is accurate. |  |
| --- | --- |
| Funded studies  Enter a statement with the following details:   - Initials of the authors who received each award - Grant numbers awarded to each author - The full name of each funder - URL of each funder website - Did the sponsors or funders play any role in the study design, data collection and analysis, decision to publish, or preparation of the manuscript? |  |
| Did you receive funding for this work? |  |
| Please add funding details.  as follow-up to "Financial Disclosure  Enter a financial disclosure statement that describes the sources of funding for the work included in this submission. Review the [submission guidelines](http://journals.plos.org/plosone/s/submission-guidelines#loc-financial-disclosure-statement) for detailed requirements. View published research articles from [*PLOS ONE*](https://journals.plos.org/plosone/) for specific examples.  This statement is required for submission and will appear in the published article if the submission is accepted. Please make sure it is accurate. | JSPS KAKENHI (grant numbers 22K15191 to SI), the Cooperative Research Program of the Wildlife Research Center, Kyoto University (2020-B-05 to SI) |

| Funded studies  Enter a statement with the following details:   - Initials of the authors who received each award - Grant numbers awarded to each author - The full name of each funder - URL of each funder website - Did the sponsors or funders play any role in the study design, data collection and analysis, decision to publish, or preparation of the manuscript? |  |
| --- | --- |
| Did you receive funding for this work?" |  |
| Please select the country of your main research funder (please select carefully as in some cases this is used in fee calculation).  as follow-up to "Financial Disclosure  Enter a financial disclosure statement that describes the sources of funding for the work included in this submission. Review the [submission guidelines](http://journals.plos.org/plosone/s/submission-guidelines#loc-financial-disclosure-statement) for detailed requirements. View published research articles from [*PLOS ONE*](https://journals.plos.org/plosone/) for specific examples.  This statement is required for submission and will appear in the published article if the submission is accepted. Please make sure it is accurate. | JAPAN - JP |
| Funded studies  Enter a statement with the following details:   - Initials of the authors who received each award - Grant numbers awarded to each author - The full name of each funder - URL of each funder website - Did the sponsors or funders play any role in the study design, data collection and analysis, decision to publish, or preparation of the manuscript? |  |
| Did you receive funding for this work?" |  |
| Competing Interests | The authors have declared that no competing interests exist. |

| Use the instructions below to enter a competing interest statement for this submission. On behalf of all authors, disclose any [competing interests](http://journals.plos.org/plosone/s/competing-interests) that could be perceived to bias this work—acknowledging all financial support and any other relevant financial or non- financial competing interests.  This statement is required for submission and will appear in the published article if the submission is accepted. Please make sure it is accurate and that any funding sources listed in your Funding Information later in the submission form are also declared in your Financial Disclosure statement.  View published research articles from  [*PLOS ONE*](http://journals.plos.org/plosone/) for specific examples. |  |
| --- | --- |
| NO authors have competing interests  Enter: *The authors have declared that no competing interests exist.*  Authors with competing interests  Enter competing interest details beginning with this statement:  *I have read the journal's policy and the authors of this manuscript have the following competing interests: [insert competing interests here]* |  |
| * typeset |  |
| Ethics Statement  Enter an ethics statement for this submission. This statement is required if the study involved:   - Human participants - Human specimens or tissue - Vertebrate animals or cephalopods - Vertebrate embryos or tissues | This study was conducted in accordance with the American Society of Primatologists Code of Best Practices for Field Primatology. Furthermore, this study conformed to the Guidelines for Field Research established by the Ethics Committee of the Primate Research Institute of Kyoto University. All methods were performed non-invasively. |

| - Field research   Write "N/A" if the submission does not require an ethics statement.  General guidance is provided below. Consult the [submission guidelines](http://journals.plos.org/plosone/s/submission-guidelines#loc-guidelines-for-specific-study-types) for detailed instructions. Make sure that all information entered here is included in the Methods section of the manuscript. |  |
| --- | --- |

| Format for specific study types  Human Subject Research (involving human participants and/or tissue)   - Give the name of the institutional review board or ethics committee that approved the study - Include the approval number and/or a statement indicating approval of this research - Indicate the form of consent obtained (written/oral) or the reason that consent was not obtained (e.g. the data were analyzed anonymously)   Animal Research (involving vertebrate animals, embryos or tissues)   - Provide the name of the Institutional Animal Care and Use Committee (IACUC) or other relevant ethics board that reviewed the study protocol, and indicate whether they approved this research or granted a formal waiver of ethical approval - Include an approval number if one was obtained - If the study involved *non-human primates*, add *additional details* about animal welfare and steps taken to ameliorate suffering - If anesthesia, euthanasia, or any kind of animal sacrifice is part of the study, include briefly which substances and/or methods were applied   Field Research  Include the following details if this study involves the collection of plant, animal, or other materials from a natural setting:   - Field permit number - Name of the institution or relevant body that granted permission |  |
| --- | --- |
|  |  |
| Data Availability  Authors are required to make all data underlying the findings described fully available, without restriction, and from the time of publication. PLOS allows rare exceptions to address legal and ethical concerns. See the [PLOS Data Policy](http://journals.plos.org/plosone/s/data-availability) and [FAQ](http://journals.plos.org/plosone/s/data-availability#loc-faqs-for-data-policy) for detailed information. | Yes - all data are fully available without restriction |

| A Data Availability Statement describing where the data can be found is required at submission. Your answers to this question constitute the Data Availability Statement and will be published in the article, if accepted. |  |
| --- | --- |
| Important: Stating ‘data available on request from the author’ is not sufficient. If your data are only available upon request, select ‘No’ for the first question and explain your exceptional situation in the text box. |  |
| Do the authors confirm that all data underlying the findings described in their manuscript are fully available without restriction? |  |
| Describe where the data may be found in full sentences. If you are copying our sample text, replace any instances of XXX with the appropriate details.   - If the data are held or will be held in a public repository, include URLs, accession numbers or DOIs. If this information will only be available after acceptance, indicate this by ticking the box below. For example: *All XXX files are available from the XXX database (accession number(s) XXX, XXX.*). - If the data are all contained within the manuscript and/or Supporting Information files, enter the following: *All relevant data are within the manuscript and its Supporting Information files.* - If neither of these applies but you are able to provide details of access elsewhere, with or without limitations, please do so. For example:   *Data cannot be shared publicly because of [XXX]. Data are available from the XXX Institutional Data Access / Ethics Committee (contact via XXX) for researchers who meet the criteria for access to confidential data.*  *The data underlying the results presented in the study are available from (include the name of the third party* | All relevant data are within the manuscript and its Supporting Information files. |

| *and contact information or URL).*   - This text is appropriate if the data are owned by a third party and authors do not have permission to share the data.   * typeset |  |
| --- | --- |
| Additional data availability information: |  |

Manuscript	[Click here to access/download;Manuscript;Manuscript.docx](https://www2.cloud.editorialmanager.com/pone/download.aspx?id=36771354&guid=058317dc-692b-42e5-a949-429a35c45351&scheme=1)[<https://www2.cloud.editorialmanager.com/pone/download.aspx?id=36771354&guid=058317dc-692b-42e5-a949-429a35c45351&scheme=1>](https://www2.cloud.editorialmanager.com/pone/download.aspx?id=36771354&guid=058317dc-692b-42e5-a949-429a35c45351&scheme=1)

# 1 Full title

2 Paternity success for resident and non-resident males and their influences on paternal sibling
3 cohorts in Japanese macaques on Shodoshima Island

4

# 5 Short title

6 Paternity for male Japanese macaques on Shodoshima Island

7

# 8 Author names and affiliations

9 Shintaro Ishizuka^1,2,3,4^, Eiji Inoue^2^, Yuki Kaji^5^
10 1. Faculty of Life Science and Technology, Fukuyama University, Fukuyama, Hiroshima
11 729-0292, Japan
12 2. Department of Biology, Faculty of Science, Toho University, 2-2-1 Miyama, Funabashi,
13 Chiba 274-8510, Japan
14 3. Japan Society for Promotion of Science, Kojimachi Business Center Building, 5-3-1
15 Kojimachi, Chiyoda-ku, Tokyo 102-0083, Japan
16 4. Primate Research Institute, Kyoto University, 41-2 Kanrin, Inuyama, Aichi 484-8506, Japan
17 5. Choshikei Monkey Park, 3387-10, Kaerugoike, Hitoyama, Tonosho, Kagawa, 761-4151,
18 Japan

19

# 20 Corresponding author

21 Shintaro Ishizuka [(ishizuka.shintaro@fukuyama-u.ac.jp)](mailto:(ishizuka.shintaro@fukuyama-u.ac.jp)

22

# 23 Author contributions

24 SI designed this study, collected behavioral and genetic data, analyzed all data, and
25 wrote the manuscript. EI supported to design this study. YK contributed to collect demographic
26 data on the study subjects. All authors gave final approval for publication.

27

# 28 Abstract

29 Reproductive success can be attributed to both resident and non-resident males in
30 non-human primates. However, reproductive success of non-resident males has rarely been
31 investigated at an individual level. As resident males achieve different degree of reproductive
32 success with regard to various factors, such as male dominance relationships or female mate
33 choice, the degree of reproductive success for non-resident males may vary between individuals.
34 As male reproductive success is highly skewed towards specific individuals, the percentage of
35 similar-aged paternal siblings within groups is expected to increase. However, the extent to
36 which each male contributes to the production of cohorts of paternal siblings remains unclear.
37 Here we examined the paternity of offspring born over five consecutive years in a free-ranging
38 group of Japanese macaques *Macaca fuscata* on Shodoshima Island, Kagawa Prefecture.
39 Genotypes of 87 individuals at 16 autosomal microsatellite loci were analyzed and paternity of
40 34 offspring was successfully assigned to a single candidate father. We quantitatively assessed
41 paternity success for resident and a few non-resident males whose genetic samples were
42 successfully collected. We quantitatively assessed the percentages of paternal siblings in the
43 same age cohorts produced by those males. Non-resident males sired similar percentage of
44 offspring compared to resident males. A large prime-aged non-resident male was the most
45 successful sire among males in two of the five years. These results provide new insights that
46 male reproductive success could be highly skewed toward a specific non-resident male.
47 Subadult males had a lower percentage of paternity success, which may be because females may
48 prefer physically mature males. Various males, including non-resident males, contributed to the
49 creation of paternal sibling in the same age cohort. The overall results highlighted that not only
50 resident but also non-resident males play an important role in shaping within-group kin
51 structures.

52

53

# 54 Introduction

55 Reproductive access to females is one of the most important issues for male animals [1]. Males
56 compete with each other for breeding opportunities [2]. As a consequence of male–male
57 competition, males often achieve different degrees of reproductive success. In group-living
58 primates, variance in reproductive success among males is sometimes explained by dominance relationships
59 [3–6]. Studies have shown that reproductive success is often skewed toward high-ranking males
60 within multimale groups [7–13]. Beside dominance relationships, female mate choice also can
61 affect the distribution of male reproductive success [14,15]. Females may prefer to mate with particular categories of males, depending on the characteristics of males, such
62 as their maturity, capacity for investment, or genetic background [16,17]. Males that are selected
63 by females can achieve high reproductive success [18–22].
64 Variance in male reproductive success has been investigated within animal
65 Groups, but, reproductive success for males outside a group has been infrequently examined. In
66 group-living animals, especially primates, both resident and non-resident males have
67 opportunities to breed with females of the group. The paternity of offspring assigned to males
68 outside a group is termed extra-group paternity (EGP). EGP is a phenomenon widely observed
69 in various primate species [23–28] and is essential for the evolution of social systems in animals
70 as it increases genetic diversity within groups and is consequently associated with the increased
71 chance of offspring survivorship [29]. EGP has been usually determined when the paternity of offspring is
72 unsuccessfully assigned to all candidate fathers within a group, probably because individual
73 identification and sample collection for non-resident males are usually difficult in the field.
74 Several studies that have examined the overall percentages of EGP have shown that their
75 percentages would increase when the number of males within groups decreases or when female
76 reproductive synchrony increases [26,30,31]. However, few studies have assigned EGP to a
77 particular non-resident male, and the number of cases in which paternity success for each
78 non-resident male has been individually is scarce [32]. Similar to the pattern
79 in resident males, the degree of reproductive success for each non-resident male may vary
80 depending on various factors, such as their dominance relationships or female choice. To
81 understand mechanisms that influence the variance in the male reproductive success among all
82 types of males, it is necessary to assess the reproductive success for not only the resident males,

but also the non-resident males, by individually identifying and sampling both.

83 Variance in the male reproductive success modulates the abundance of paternal kin living in
84 animal groups. The presence of kin within groups plays important roles for their social lives
85 because kinship is one of the key factors that affects affinity among individuals, and reciprocity
86 and cooperation are attributed to kin selection [33–35]. In primates, evidence of paternal kin
87 bias behavior has been accumulated [36–39]. The evidence suggests that primate individuals
88 have more social partners as the number of paternal kin increases in their groups. Theoretically,
89 the percentage of paternal sibling dyads in the same age cohort is expected to increase with a
90 higher male reproductive skew [40–42]. Since male reproductive success is often skewed
91 toward the highest-ranking males in primate groups, researchers have assessed the percentage of
92 paternal sibling dyads in the same age cohort according to the percentage of paternity success
93 by the highest-ranking male [38,42,43]. However, the paternity success can be skewed towards
94 other resident males or non-resident males besides the highest-ranking male. The contributions
95 of lower ranked males to the percentages of paternal sibling dyads in the same age
96 cohorts have not been investigated. For a comprehensive understanding of the mechanisms that
97 produce kin-dyads within animal groups, it is crucial to examine the percentages of paternal sibling dyads
98 produced by non-resident males at an individual level.
99 To investigate the paternity success for non-resident males and their contributions to
100 the production of kin-dyads within groups, the Japanese macaque *Macaca fuscata* is an
101 interesting species. They form multi-male and multi-female bisexual groups, in which females
102 are philopatric and form strong affiliative relationships with matrilineal female kin [44–51].
103 Males typically emigrate from their natal group when they are approximately four or five years
104 old [52] and continue to transfer between groups throughout life [53]. The tenure of males in a
105 group is approximately three years [54]. Dominance rank is linear and stable in both
106 sexes [54–56], although troop takeovers by non-troop males were reported from the Yakushima
107 population [57]. Notably, non-resident males often breed with females of the group, as the
108 percentages of EGP are relatively high among primates [28,31]. This is partially because they
109 have breeding seasonality [58,59], and novel and unfamiliar males attain high reproductive
110 success because they are strongly selected by females for mating partners when females are
111 likely to conceive [19,21,60,61]. Further, the exact number of siring for each non-resident male
112 in the field can also be assessed. During the mating season, several non-resident males
113 sporadically appear around the group and copulate with the group’s females [24,62].
114 Considering that previous field research succeeded in collecting behavioral data and genetic
115 samples from non-resident males [19,24,63], it is possible to expand on it further to examine the
116 paternity success for non-resident males at the individual level. Moreover, the percentage of
117 paternity by the highest-ranking male is usually low [19,24,64], although one study reported a
118 relatively high percentage [65]. This suggests that the percentages of paternal sibling dyads
119 produced by non-resident males can be assessed quantitatively.
120 In this study, we examined the paternity of the offspring born in five consecutive years
121 within a group of Japanese macaques, wherein resident and a few of non-resident males were
122 identified and sampled. We assessed the percentages of paternity success for both resident and
123 non-resident males and the percentages of paternal sibling dyads produced by them among the

127

128

offspring in each year.

# 129 Materials and Methods

130 **Study subjects**
131 The study subjects were free-ranging Japanese macaques of the B-group inhabiting an
132 area near the Choshikei Monkey Park on Shodoshima Island [51]. The macaques are
133 provisioned by park staff at approximately 8:30 h, 14:00 h, and 16:00 h and by tourists visiting
134 the park. We initially conducted field research on the subjects and started individual
135 identification of the group between September and December 2017. In April 2018, we started
136 monitoring the presence of identified individuals in the monkey park on a daily basis (five or six
137 days per week), although their presence has not been quantitatively recorded. In February 2019,
138 we successfully completed individual identification of group members, and started systematically
139 recording the daily presence of the members of the group. The mean number of observation
140 days per month for 2019–2022 was 25.6. The group size, the number of adult females, and the

141

142

143

number of adult and subadult males for 2019–2021 are shown in Table 1.

# 144 Table 1. Composition of the B-group at the end of 2019–2021.

|  | 2019 | 2020 | 2021 |
| --- | --- | --- | --- |
| Adult females | 29 | 16 | 16 |
| Adult and subadult males | 12 | 13 | 11 |
| Juveniles and infants | 40 | 35 | 33 |
| Group size | 81 | 64 | 60 |

145 The number of group members gradually decreased from 2019 to 2020. Although the reason for
146 the decline remains unclear, this might be because several members were captured by the

147

148

149

government or local people.

# 150 Classification of males

151 This study focused on males estimated to be aged ≧ 4 years and sexually mature [66].
152 They were classified based on their age class and social status. Following a previous study [19],
153 the age class of males aged 4–6 years and that > 6 years was categorized as “subadult” and
154 “adult,” respectively.
155 For this study, the males were first divided into resident males of the study group or
156 non-resident males according to the daily monitoring. After completing individual identification
157 of the study group in February 2019, we sometimes encountered unidentified males around the
158 mating season. Those males were seemingly unhabituated to human observers and did not eat
159 provisioned food, implying that they were not members of the group. We regarded those males
160 as “non-resident males”. We identified four non-resident males and three of them were
161 successfully sampled.
162 The social status of the resident males was further divided into dominant or
163 subordinate males. Males of provisioned groups constantly compete with one another over food,
164 which emphasizes the dominance-subordinate aspects of their relationships [67]. In our study,
165 some males were allowed to access preferred food at the provisioning area, whereas other males
166 were driven away by both the males and females who stayed at the provisioning area and were
167 thus uncommonly observed to enter into the area. Therefore, we classified those males that were
168 allowed to access the provisioning area as dominant and those that were driven away from the
169 provisioning area as subordinate males. Collectively, the social status of males was classified
170 into three categories: dominant, subordinate, and non-resident males. This is in accordance with
171 previous studies on macaque species [24,68]. Two to three dominant, eight to ten subordinate,
172 and two identified non-resident males were present between 2019 and 2021, respectively. The
173 presence and social/age class of all identified males in each mating/non-mating season is shown
174 in Table S1. We regarded September–December as the mating season, and January–August as
175 the non-mating season because births occur between March and June [58,59], and the

176

177

mean gestation length of this species is 176 days [69].

# 178 Genetic analysis

179 We used genotype data for the study subjects at 16 autosomal microsatellite loci,
180 which were obtained in our previous work [51]. Feces, sperm, and saliva samples were collected
181 using cotton swabs and stored in lysis buffer at ambient temperature immediately after we
182 observed defecation, ejaculation, and materials the animals sucked in the field. DNA was
183 extracted from the samples using a QIAamp Stool Mini Fast Kit (Qiagen, CA, USA). Using
184 DNA extracts, the genotypes at 16 autosomal microsatellite loci were analyzed using the
185 multiplex PCR method [70]. Amplification products were separated by capillary electrophoresis
186 using an ABI 3130xl Genetic Analyzer (Applied Biosystems, CA, USA). Alleles were sized
187 using Peak Scanner (Applied Biosystems). Since DNA extracted from non-invasively collected
188 samples is typically degraded and low in concentration [71,72], we repeated the genotyping to
189 ensure accuracy following the criteria in the previous study [70]. Details for the methods of

190

191

genetic analysis are described in a previous study [51].

# 192 Paternity analysis

193 The paternity of 46 offspring (8 of 18 in 2018, 9 of 19 in 2019, 8 of 13 in 2020, 10 of
194 10 in 2021, and 11 of 11 in 2022, respectively) was investigated. The offspring whose paternity
195 was not investigated were already missing and no genetic samples had been collected from them.
196 Paternity analysis was conducted using the pairwise likelihood approach with CERVUS [73].
197 The resident candidate fathers were only fully sampled for the offspring born in 2020, 2021, and
198 2022. Therefore, the percentage of EGP could be calculated for only these years. According to
199 our field observations, there seemed to be approximately 10 unsampled candidate fathers who
200 belonged to the group during the 2017 and/or 2018 mating seasons or were non-resident males.
201 Since we collected genetic samples for 19 candidate fathers, which accounted for 60–70% of all
202 candidate fathers, the proportion of sampled candidate fathers was eventually assumed to be
203 0.60. The proportion of loci mistyped and the error rate in the likelihood calculation was both
204 0.01 because genotype accuracy was confirmed following reasonable criteria [70]. Paternity for
205 42 of the 46 offspring was analyzed under the condition that the offspring’s maternity was
206 known since genotypes for the mothers were determined. However, genetic samples for mothers
207 of four offspring could not be collected. Therefore, the paternity for the four offspring was
208 analyzed under the condition that the maternity of the offspring was unknown. When the most
209 likely father had no mismatched alleles and the confidence level for the assignments was more
210 than 95%, the male was concluded to be the offspring’s father.

226

| 211 |  |
| --- | --- |
| 212 | **Statistical analysis** |
| 213 | To analyze the likelihood of siring by each male, we constructed generalized linear |
| 214 | mixed models (GLMMs) with binomial distribution and logit link function. We included the |
| 215 | percentage of sires for each male in each year as a response variable. Since the total number of |
| 216 | sampled males was 10, 10, 12, 12, and 13 in 2017, 2018, 2019, 2020, and 2021, respectively, the |
| 217 | total number of data points was 57. The response variable ranged from 0 to 0.40, as the most |
| 218 | successful sire’s share in a year was 40% (see the Results). The social status |
| 219 | (dominant/subordinate/non-resident) and age class (adult/subadult) of each male and the number |
| 220 | of newborns, which corresponds to the approximate number of estrus capable females in each |
| 221 | year (18 in 2018, 19 in 2019, 13 in 2020, 10 in 2021, and 11 in 2022), were included as |
| 222 | predictor variables. Offspring ID and the study year were included as random effects. We |
| 223 | calculated the Akaike’s information criterion (AIC) for the constructed model and null model. |
| 224 | Comparing the AIC values, we assessed the fit of constructed model. The models were fit using |
| 225 | the “lmer” function of the R package lme4 [74]. |
| 227 | **Ethics statement** |
| 228 | This study was permitted by the Choshikei Monkey Park on Shodoshima Island, Japan. |
| 229 | All methods were designed to be noninvasive for the subjects. Field research was conducted in |
| 230 | accordance with the American Society of Primatologists Code of Best Practices for Field |
| 231 | Primatology, and conformed to the Guidelines for Field Research established by the Ethics |
| 232 | Committee of the Primate Research Institute of Kyoto University. All aspects of this study |
| 233 | adhered to the ASAB/ABS Guidelines for the use of animals in research. |

234

| 235 | **Results** |
| --- | --- |
| 236 | **Paternity** |
| 237 | Genotypes of the 88 individuals at 16 autosomal microsatellite loci were analyzed |
| 238 | (Table S2). The number of alleles, heterozygosity, and allelic dropout rate are investigated based |
| 239 | on the genotypes (Table S3). The paternity of 34 of the 46 offspring was assigned to a single |
| 240 | candidate father (Fig. 1 and Table S4). At least one offspring was sired by sampled non-resident |
| 241 | males in each year. Since all resident candidate fathers for offspring born in 2018 and 2019 were |
| 242 | not sampled, it remains unclear whether or not these offspring were sired by non-resident males, |
| 243 | and thus, the percentage of EGP could not be calculated for those years. However, for the years |
| 244 | in which all resident candidate fathers were sampled, the results revealed that the percentage of |
| 245 | EGP was 50% (4 of 8) in 2020, 50% (5 of 10) in 2021, and 9% (1 of 11) in 2022. The overall |
| 246 | percentage of EGP between 2020 and 2022 was 34% (10 of 29). Interestingly, 75% (3 of 4) and |
| 247 | 80% (4 of 5) of the EGP cases were assigned to a single non-resident male “NP” in 2020 and |
| 248 | 2021, respectively. He was the most successful sire and sired 38% (3 of 8) and 40% (4 of 10) of |
| 249  250 | offspring in 2020 and 2021, respectively. |
| 251  252 | **Fig. 1. The number of offspring sired by each male in the five-year study period.** |
| 253 | Gray, lined, and dot bars indicate sires by the dominant, subordinate, and non-resident males, |
| 254 | respectively. White bars indicate sires whose father’s social status remained unclear. The ID of |
| 255 | all fathers is shown in each bar. The values within brackets represent the number of paternities |
| 256 | analyzed in each year. |

257

258

| 259 | **Effects of male categories on the percentages of paternity success** | | | | | |
| --- | --- | --- | --- | --- | --- | --- |
| 260 | The constructed model was significantly better fit than the null model (ΔAIC = 10.99, | | | | | |
| 261 | P < 0.001). The constructed model showed that the effects of neither male social status nor the | | | | | |
| 262 | number of capable females were significant (Fig. 2A and Table 2). The effect of male age class | | | | | |
| 263 | had a negative effect on the percentage of paternity success, indicating that paternity success of | | | | | |
| 264  265 | subadult males was lower than that of the adult males (Fig. 2B and Table 2). | | | | | |
| 266  267 | **Table 2. Results of the generalized linear mixed model (GLMM) performed to test the** | | | | | |
| 268 | **effects of males’ social status and their age class.** | | | | | |
|  | Parameter | Log-Odds | SE | 95% CI | Z | P |
|  | (Intercept) | −1.60 | 1.10 | −3.76 to 0.55 | −1.46 | 0.145 |
|  | Male category (non-resident | 0.47 | 1.09 | −1.67 to 2.60 | 0.43 | 0.669 |
|  | vs. dominant) |  |  |  |  |  |
|  | Male category (non-resident | 0.11 | 0.75 | −1.37 to 1.58 | 0.14 | 0.888 |
|  | vs. subordinate) |  |  |  |  |  |
|  | Age (adult vs. subadult) | −3.00 | 1.05 | −5.06 to −0.95 | −2.86 | < 0.01 |
|  | No. of capable females | −0.08 | 0.06 | −0.21 to 0.04 | −1.26 | 0.208 |
| 269 |  |  |  |  |  |  |
| 270 |  |  |  |  |  |  |
| 271 |  |  |  |  |  |  |

# 272 Fig. 2. The effects of males’ social status and their age class on their paternity success.

273 Percentage of paternity success by each male in each year according to the males’ social status
274 (A) and their age classes (B) are shown. Boxes indicate the first to third quartile of observed
275 values, solid lines show the median, and each dot represents the percentage of paternity success

276

277

278

by a sampled candidate father in each year.

# 279 The proportion of paternal sibling dyads

280 The percentages of paternal sibling dyads among the offspring born in 2018, 2019,
281 2020, 2021, and 2022 were 0%, 8%, 14%, 27%, and 16%, respectively (Table 3). The overall
282 percentage of paternal sibling dyads was 15%. Interestingly, subordinate males or

283

284

285

non-resident males produced 11–13% of paternal sibling dyads between 2020 and 2022.

286 **Table 3. The percentage of paternal sibling dyads among offspring born in each year. The**
287 **values within the brackets represent the number of paternal sibling dyads in each**
288 **category.**

| Year | No. of dyads analyzed | Percentage of paternal sibling  dyads | Maximum percentage of paternal sibling dyads produced by one  male | Male category of the most  successful sire |
| --- | --- | --- | --- | --- |

| 2018 | 28 | 0(0)* | N/A | N/A |
| --- | --- | --- | --- | --- |
| 2019 | 36 | 8(3)* | N/A | N/A |
| 2020 | 28 | 14(4) | 11(3) | NR |
| 2021 | 45 | 27(12) | 13(6) | S and NR |

| 2022 | 55 | 16(9) | 11(6) | S |
| --- | --- | --- | --- | --- |
| Total | 192 | 15(28) | 12(15) | - |

289 S and NR represent subordinate and non-resident males, respectively. N/A represents data that
290 was not available.
291 * The percentages may have been underestimated because offspring whose fathers remain

292

293

294

unidentified may have been paternal siblings.

# 295 Discussion

296 The percentage of EGP between 2020 and 2022 was 34% (9–50%) in the Shodoshima
297 B-group. These results are consistent with previous results showing that 23–80% of offspring
298 were sired by non-resident males (Table 4). The percentage of EGP in this species is relatively
299 high among primate species [28,31]. One reason for the relatively high percentage of EGP may
300 be the high extent of female receptive synchrony in this species [65]. EGP is more likely when
301 the number of males within groups decreases and when female reproductive synchrony
302 increases [26,30,31]. Similar to other species that exhibit both high female receptive synchrony

303

304

and EGP, resident males may not guard receptive females against non-resident males.

# 305 Table 4. Overview paternity results in Japanese macaques.

Site	Number

Number

Condition	of

Period	Number

Maximum

Note	for	the

Overall

Reference

| of adult | of adult | study site |  | of | percentage | status of the most | EGP (%) |  |
| --- | --- | --- | --- | --- | --- | --- | --- | --- |
| males | females |  |  | paternity | of paternity | successful sire |  |  |
|  |  |  |  |  | for one male |  |  |  |
|  |  |  |  |  | (%) |  |  |  |
| Kyoto  19  University | 30 | Captive | 1988 | 48 | 22.9 | Highest-ranking | - | [64] |
| Yakushima,  15 | 15 | Wild | 1998 | 9 | 22.2^b^ | Highest-ranking | 33 | [65] |
| Nina-A |  |  |  |  |  |  |  |  |
| Yakushima, |  |  | 1999– |  |  | Low-ranking, |  |  |
| 7–10  Nina-A | 2–7 | Wild | 2000 | 4 | 25 | and non-resident | 25 |  |
|  |  |  |  |  |  |  |  | [24] |
| Yakushim, B 1–7 | 6–7 | Wild | 1996– | 5 | 20^b^ | High-ranking, | 80 |  |
|  |  |  | 2000 |  |  | low-ranking, and |  |  |

non-resident

Arashiyama	24–27	93–100	Free-ranging

2002–

2003

23	15.3	Low-ranking	23	[19]

Oregon	17	N/A

Semi

free-ranging

2018–

2019

34	20.5	High-ranking^c^	-	[21]

Shodoshima^a^	11–13	16–29	Free-ranging

2019–

2021

46	27.5	Non-resident	34

Present study

306 a The data in 2018 and 2019 were excluded because of incomplete individual identification.
307 b The percentage may become higher if EGP is attributed to the single male.

308

309

c It remains unclear whether the male was the highest-ranking male.

310 A non-resident male, “NP,” was the most successful sire in both 2020 and 2021. Our
311 results highlighted that at least some proportion of non-resident males could have chances to
312 gain a high percentage of paternity success, although the reproductive success of non-resident
313 males as a whole cannot be assessed because of the incomplete sampling of non-resident males.
314 Given that the non-resident males sampled in this study might have chances to breed with
315 females of other groups beside our study group, the percentage of paternity success for these
316 non-resident males might have been underestimated. Since female Japanese macaques prefer to
317 mate with unfamiliar males [75,76], non-resident males may maintain unfamiliarity with
318 females for at least several years, and consequently have several chances to breed with females.
319 Furthermore, our results provide new insights that there is a reproductive skew among
320 non-resident males: 75–80% of EGP were attained by “NP” in 2020 and 2021, while high ranking resident “TN” was
321 unsuccessful in siring offspring in those two years. According to our field observations, “NP”
322 was a large prime-aged male and displayed frequently (Fig. 3). A previous study in captivity
323 showed that female Japanese macaques expressed mate choice behavior toward males who
324 displayed most frequently when they were likely to conceive [61]. Since non-resident males are
325 typically unfamiliar to females and the extent of unfamiliarity to females might not differ
326 between them, a non-resident male who displayed frequently might be favored as females’

327

328

329

330

mating partner and gain higher paternity success.

# 331 Fig. 3. A photograph of the non-resident male who attained a high percentage of paternity

332

333

**success.** His individual ID was “NP”.

334

335 Notably, subadult males sired a lower percentage of offspring than the adult males.
336 These results were consistent with previous studies showing that males reach high reproductive
337 success when they are approximately six years old in rhesus macaques *Macaca mulatta* [77,78],
338 and subadult males (aged 4.5–6.5 years old) had a much lower reproductive success than adult
339 males (7.5–25 years old) in Barbary macaques *Macaca sylvanus* [79]. The tendency in which
340 males who reach an age of approximately six years old can gain high amounts of reproductive
341 success may be common among macaque species, although all of these results were observed in
342 provisioned or semi-closed environments. Age-related variance in male reproductive success
343 may be influenced by a function of the interaction between social skills and morphological traits
344 [80,81], or facial coloration of males [82–84].
345 The percentage of paternal sibling dyads among offspring in the same age cohort was
346 15% (8–27%) in the Shodoshima-B group. Although available data for comparisons with our
347 results is scarce, the percentage is relatively high compared to 12% in rhesus macaques [42] and
348 5% in Assamese macaques *Macaca assamensis* [38]. As shown in Table 4, the most successful
349 sires were the highest-ranking males in only two or three of the seven cases. These results
350 suggest that various males, including non-resident males, contribute significantly to the
351 production of paternal sibling dyads in Japanese macaques. The presence of paternal sibling
352 dyads in the same age cohort may trigger kin selection and enhance affinity among individuals
353 of same age. In female-philopatric primates, females of same or similar age often form strong
354 affiliative relationships (e.g. brown capuchin monkeys *Cebus apella nigritus*: [85]; chacma
355 baboons *Papio hamadryas ursinus*: [86]; rhesus macaques: [87]). In Japanese macaques on the
356 Katsuyama population, the two subject females of the same age formed long-term grooming
357 partnerships [49]. Such an affinity among same or similar-aged individuals in Japanese
358 macaques may be favored by paternal sibling relationships, although it is unclear whether
359 individuals can discriminate paternal kin. Future behavioral and genetic studies are required to
360 clarify the presence or absence of paternal kin bias in this species.

Our findings contribute to a better understanding of how male social status influences diverse reproductive strategies. Notably, this study clearly showed that at least some

361 non-resident males can have high reproductive success. Furthermore, various males,
362 including non-resident males, significantly contribute to the production of paternal sibling dyads
363 in the same age cohorts, suggesting that not only the resident males, but also non-resident males
364 play an important role in shaping within-group kin structures. However, future studies are

367

368

required to examine how paternal siblings interact with each other.

# 369 Acknowledgments

370 We thank Dr. K. Watanabe, Mr. A. Nishio, Ms. C. Saeki, Mr. M. Ishii, Ms. K.
371 Miyashita, and Mr. K. Hida for their help with the fieldwork. This study was financially
372 supported by the Japan Society for the Promotion of Science Grant-in-Aid for JSPS fellows
373 (21J00922 to SI), Cooperative Research Program of the Wildlife Research Center, Kyoto
374 University (2020-B-05 to SI), and Leading Graduate Program in Primatology and Wildlife
375 Science	of Kyoto	University.	English	language	editing was	provided by Editage

376

377

(https://[www.editage.jp).](http://www.editage.jp/)

378	**References**

379	1.	Trivers R. Parental investment and sexual selection. In: Campbell B, editor. Sexual

380	Selection and the Descent of Man. New York: Aldine de Gruyter; 1972. pp. 136–179.

381	2.	van Noordwijk MA, van Schaik CP. Sexual selection and the careers of primate males:

382	paternity concentration, dominance-acquisition tactics and transfer decisions. In: Kappeler

383	PM, van Schaik CP, editors. Sexual Selection in Primates. Cambridge: Cambridge

384	University Press; 2004. pp. 208–229.

385	3.	Bernstein IS. Dominance, aggression and reproduction in primate societies. J Theor Biol.

386	1976;60: 459–472.

387	4.	Cowlishaw G, Dunbar RIM. Dominance rank and mating success in male primates. Anim

388	Behav. 1991;41: 1045–1056.

389	5.	De Ruiter JR, Van Hooff JA. Male dominance rank and reproductive success in primate

390	groups. Primates. 1993;34: 513–523.

391 6.	Kutsukake N, Nunn CL. The causes and consequences of reproductive skew in male
392 primates. In: Hager R, Jones CB, editors. Reproductive skew in vertebrates: proximate
393 and ultimate causes. Cambridge: Cambridge University Press; 2009. pp. 165–195.
394 7.	Girman DJ, Mills MGL, Geffen E, Wayne RK. A molecular genetic analysis of social
395 structure, dispersal, and interpack relationships of the African wild dog (*Lycaon pictus*).
396 Behav Ecol Sociobiol. 1997;40: 187–198.
397 8.	Kutsukake N, Nunn CL. Comparative tests of reproductive skew in male primates: The
398 roles of demographic factors and incomplete control. Behav Ecol Sociobiol. 2006;60: 695–
399 706.
400 9.	Engelhardt A, Heistermann M, Hodges JK, Nürnberg P, Niemitz C. Determinants of male
401 reproductive success in wild long-tailed macaques (*Macaca fascicularis*)—male
402 monopolisation, female mate choice or post-copulatory mechanisms?. Behav Ecol
403 Sociobiol. 2006;59: 740–752.
404 10. Inoue E, Inoue‐Murayama M, Vigilant L, Takenaka O, Nishida T. Relatedness in wild
405 chimpanzees: influence of paternity, male philopatry, and demographic factors. Am J Phys
406 Anthropol. 2008;137: 256–262.
407 11. Willisch CS, Biebach I, Koller U, Bucher T, Marreros N, Ryser-Degiorgis MP, et al. Male
408 reproductive pattern in a polygynous ungulate with a slow life-history: the role of age,
409 social status and alternative mating tactics. Evol Ecol. 2012;26: 187–206.
410 12. Ishizuka S, Kawamoto Y, Sakamaki T, Tokuyama N, Toda K, Okamura H, et al. Paternity
411 and kin structure among neighbouring groups in wild bonobos at Wamba. R Soc Open Sci.
412 2018;5: 171006.
413 13. Higham JP, Heistermann M, Agil M, Perwitasari-Farajallah D, Widdig A, Engelhardt A.
414 Female fertile phase synchrony, and male mating and reproductive skew, in the crested
415 macaque. Sci Rep. 2021;11: 4251.
416 14. Poole JH. Mate guarding, reproductive success and female choice in African elephants.

417	Anim Behav. 1989;37: 842–849.

418 15. Drickamer LC, Gowaty PA, Holmes CM. Free female mate choice in house mice affects
419 reproductive success and offspring viability and performance. Anim Behav. 2000;59: 371–
420 378.
421 16. Small ME. Female choice in nonhuman primates. Am J Phys Anthropol. 1989;32: 103–127.
422 17. Clutton-Brock TH, McAuliffe K. Female Mate Choice in Mammals. Q Rev Biol. 2009;84:

423	3–27.

424 18. Radespiel U, Dal Secco V, Drögemüller C, Braune P, Labes E, Zimmermann E. Sexual
425 selection, multiple mating and paternity in grey mouse lemurs, *Microcebus murinus*. Anim
426 Behav. 2002;63: 259–268.
427 19. Inoue E, Takenaka O. The effect of male tenure and female mate choice on paternity in
428 free-ranging Japanese macaques. Am J Primatol. 2008;70: 62–68.
429 20. Dubuc C, Muniz L, Heistermann M, Engelhardt A, Widdig A. Testing the priority-of-access
430 model in a seasonally breeding primate species. Behav Ecol Sociobiol. 2011;65: 1615–
431 1627.
432 21. Gartland KN, Biggs N, Shreeve CM, White FJ. Dominance rank, female choice, and
433 reproductive success in semi‐free ranging adult male Japanese macaques (*Macaca fuscata*).
434 Am J Primatol. 2021;83: e23294.
435 22. Schembari S, Miller C, Roberts SJ, Cords M. Female Mate Choice in Wild Kenyan Blue
436 Monkeys (*Cercopithecus mitis*). Animals, 2024;14: 1589.
437 23. Boesch C, Kohou G, Néné H, Vigilant L. Male competition and paternity in wild
438 chimpanzees of the Taï forest. Am J Phys Anthropol. 2006;130: 103–115.
439 24. Hayakawa S. Male–female mating tactics and paternity of wild Japanese macaques
440 (*Macaca fuscata yakui*). Am J Primatol. 2008;70: 986–989.
441 25. Roberts, S. J., Nikitopoulos, E., & Cords, M. (2014). Factors affecting low resident male
442 siring success in one-male groups of blue monkeys. Behavioral Ecology, 25(4), 852-861.
443 26. Ruiz-Lambides AV, Weiß BM, Kulik L, Stephens C, Mundry R, Widdig A. Long-term
444 analysis on the variance of extra-group paternities in rhesus macaques. Behav Ecol
445 Sociobiol. 2017;71: 67.
446 27. Masi S, Austerlitz F, Chabaud C, Lafosse S, Marchi N, Georges M, et al. No evidence for
447 female kin association, indications for extragroup paternity, and sex‐biased dispersal
448 patterns in wild western gorillas. Ecol Evol. 2021;11: 7634–7646.
449 28. Miller CM, Snyder-Mackler N, Nguyen N, Fashing PJ, Tung J, Wroblewski EE, et al.
450 Extragroup paternity in gelada monkeys, *Theropithecus gelada*, at Guassa, Ethiopia and a
451 comparison with other primates. Anim Behav. 2021;177: 277–301.
452 29. Reed DH, Frankham R. Correlation between fitness and genetic diversity. Conserv Biol.

453	2003;17: 230–237.

454	30. Isvaran K, Clutton-Brock T. Ecological correlates of extra-group paternity in mammals.

455	Proc R Soc Lond B*.* 2007;274: 219–224.

456 31. Ostner J, Nunn CL, Schülke O. Female reproductive synchrony predicts skewed paternity
457 across primates. Behav Ecol. 2008;19: 1150–1158.
458 32. Roberts, SJ, Cords, M. Life as a bachelor: quantifying the success of an alternative
459 reproductive tactic in male blue monkeys. PeerJ. 2015;3: e1043.
460 33. Hamilton WD. The genetical evolution of social behaviour. I. J Theor Biol. 1964;7: 1–16.
461 34. Smith JE. Hamilton’s legacy: kinship, cooperation and social tolerance in mammalian

462	groups. Anim Behav. 2014;92: 291–304.

463	35. Silk JB. Nepotistic cooperation in non-human primate groups. Phil Trans R Soc Lond B.

464	2009;364: 3243–3254.

465 36. Widdig A, Nürnberg P, Krawczak M, Streich WJ, Bercovitch FB. Paternal relatedness and
466 age proximity regulate social relationships among adult female rhesus macaques. Proc Natl
467 Acad Sci USA. 2001;98: 13769–13773.
468 37. Smith K, Alberts SC, Altmann J. Wild female baboons bias their social behaviour towards
469 paternal half-sisters. Proc R Soc Lond B. 2003;270: 503–510.
470 38. De Moor D, Roos C, Ostner J, Schülke O. Female Assamese macaques bias their affiliation
471 to paternal and maternal kin. Behav Ecol. 2020;31: 493–507.
472 39. Cords M, Minich T, Roberts SJ, Sleator C. Evidence for paternal kin bias in the social
473 affiliation of adult female blue monkeys. Am J Primatol. 2018;80: e22761.
474 40. Altmann J. Age cohorts as paternal sibships. Behav Ecol Sociobiol. 1979;6: 161–164.
475 41. Lukas D, Reynolds V, Boesch C, Vigilant L. To what extent does living in a group mean
476 living with kin? Mol Ecol. 2005;14: 2181–2196.
477 42. Widdig A. The impact of male reproductive skew on kin structure and sociality in
478 multi-male groups. Evol Anthropol. 2013;22: 239–250.
479 43. Langergraber KE, Mitani JC, Vigilant L. The limited impact of kinship on cooperation in
480 wild chimpanzees. Proc Natl Acad Sci USA. 2007;104: 7786–7790.
481 44. Koyama N. Grooming relationships in the Arashiyama group of Japanese monkeys. In:
482 Fedigan LM, Asquith PJ, editors. The monkeys of Arashiyama: Thirty-five years of
483 research in Japan and the West. New York: State University of New York Press. 1991. pp.
484 211–226.
485 45. Muroyama Y. Mutual reciprocity of grooming in female Japanese macaques (*Macaca*

486	*fuscata*). Behaviour. 1991;119: 161–170.

487 46. Takahashi H, Furuichi T. Comparative study of grooming relationships among wild
488 Japanese macaques in Kinkazan A troop and Yakushima M troop. Primates, 1998;39: 365–
489 374.
490 47. Matsumura S. The evolution of “egalitarian” and “despotic” social systems among
491 macaques. Primates. 1999;40: 23–31.
492 48. Nakamichi M, Shizawa Y. Distribution of grooming among adult females in a large,
493 free-ranging group of Japanese macaques. Int J Primatol. 2003;24, 607–625.
494 49. Nakamichi M, Yamada K. Long‐term grooming partnerships between unrelated adult
495 females in a free‐ranging group of Japanese monkeys (*Macaca fuscata*). Am J Primatol.
496 2007;69: 652–663.
497 50. Thierry B. Unity in diversity: lessons from macaque societies. Evol Anthropol. 2007;16:

498	224–238.

499 51. Ishizuka S, Inoue E. Sex-specific effects of juvenile offspring on their mothers’ social
500 relationships with other females in Japanese macaques (*Macaca fuscata*) on Shodoshima
501 Island. Int J Primatol. 2023;44: 500–516.
502 52. Matsumura S. Intergroup affiliative interactions and intergroup transfer of young male
503 Japanese macaques (*Macaca fuscata*). Primates. 1993;34: 1–10.
504 53. Sprague DS. Life history and male intertroop mobility among Japanese macaques (*Macaca*
505 *fuscata*). Int J Primatol. 1992;13: 437–454.
506 54. Suzuki S, Hill DA, Sprague DS. Intertroop transfer and dominance rank structure of
507 nonnatal male Japanese macaques in Yakushima, Japan. Int J Primatol. 1998;19: 703–722.
508 55. Eaton GG. The social order of Japanese macaques. Scient Amer. 1976;235: 96–107.
509 56. Furuichi T. Interindividual distance and influence of dominance on feeding in a natural
510 Japanese macaque troop. Primates. 1983;24: 445–455.
511 57. Hayakawa S, Soltis J. Troop takeover and reproductive success of wild male Japanese
512 macaques on Yakushima Island (*Macaca fuscata yakui*). Int J Zool. 2011;2011: 308469.
513 58. Kawai M, Azuma S, Yoshiba K. Ecological studies of reproduction in Japanese monkeys
514 (*Macaca fuscata*) I. Problems of the birth season. Primates. 1967;8: 35–73.
515 59. Fooden J, Aimi M. Birth-season variation in Japanese macaques, *Macaca fuscata*. Primates.

516	2003;44: 109–117.

517 60. Takahata Y, Huffman MA, Suzuki S, Koyama N, Yamagiwa J. Why dominants do not
518 consistently attain high mating and reproductive success: a review of longitudinal Japanese
519 macaque studies. Primates. 1999;40: 143–158.
520 61. Soltis J, Mitsunaga F, Shimizu K, Yanagihara Y, Nozaki M. Female mating strategy in an
521 enclosed group of Japanese macaques. Am J Primatol. 1999;47: 263–278.
522 62. Takahashi H. Influence of fluctuation in the operational sex ratio to mating of troop and
523 non-troop male Japanese macaques for four years on Kinkazan Island, Japan. Primates.
524 2001;42: 183–191.
525 63. Kawazoe T. Association patterns and affiliative relationships outside a troop in wild male
526 Japanese macaques, *Macaca fuscata*, during the non-mating season. Behaviour.
527 2016;153: 69–89.
528 64. Inoue M, Takenaka A, Tanaka S, Kominami R, Takenaka O. Paternity discrimination in a
529 Japanese macaque group by DNA fingerprinting. Primates. 1990;31: 563–570.
530 65. Soltis J, Thomsen R, Takenaka O. The interaction of male and female reproductive
531 strategies and paternity in wild Japanese macaques, *Macaca fuscata*. Anim Behav. 2001;62:
532 485–494.
533 66. Takahata Y, Suzuki S, Agetsuma N, Okayasu, N, Sugiura, H, Takahashi, H, et al.
534 Reproduction of wild Japanese macaque females of Yakushima and Kinkazan islands: a
535 preliminary report. Primates. 1998;39: 339–349.
536 67. Hill DA. Effects of provisioning on the social behaviour of Japanese and rhesus macaques:
537 implications for socioecology. Primates. 1999;40: 187–198.
538 68. Toyoda A, Maruhashi T, Kawamoto Y, Matsudaira K, Matsuda I, Malaivijitnond S. Mating
539 and reproductive success in free-ranging stump-tailed macaques: effectiveness of male–
540 male coalition formation as a reproductive strategy. Front Ecol Evol. 2022;10: 802012.
541 69. Fujita S, Sugiura H, Mitsunaga F, Shimizu K. Hormone profiles and reproductive
542 characteristics in wild female Japanese macaques (*Macaca fuscata*). Am J Primatol.
543 2004;64: 367–375.
544 70. Arandjelovic M, Guschanski K, Schubert G, Harris TR, Thalmann O, Siedel H, et al.
545 Two-step multiplex polymerase chain reaction improves the speed and accuracy of
546 genotyping using DNA from noninvasive and museum samples. Mol Ecol. 2009;9: 28–36.
547 71. Taberlet P, Griffin S, Goossens B, Questiau S, Manceau V, Escaravage N, Waits LP, Bouvet
548 J. Reliable genotyping of samples with very low DNA quantities using PCR. Nucleic Acids
549 Res. 1996;24: 3189–3194.
550 72. Morin PA, Chambers KE, Boesch C, Vigilant L. Quantitative polymerase chain reaction
551 analysis of DNA from noninvasive samples for accurate microsatellite genotyping of wild
552 chimpanzees (*Pan troglodytes verus*). Mol Ecol. 2001;10: 1835–1844
553 73. Marshall TC, Slate J, Kruuk LE, Pemberton JM. Statistical confidence for likelihood-based
554 paternity inference in natural populations. Mol Ecol*.* 1998;7: 639–655.
555 74. Bates D, Machler M, Bolker B, Walker S. Fitting linear mixed-effects models using lme4. J

556	Stat Softw. 2015;67: 1–48.

557	75. Huffman MA. Consort intrusion and female mate choice in Japanese macaques (*Macaca*

558	*fuscata*). Ethology. 1987;75: 221–234.

559 76. Perloe SI. Male mating competition, female choice and dominance in a free ranging group
560 of Japanese macaques. Primates. 1992;33: 289–304.
561 77. Bercovitch FB, Widdig A, Trefilov A, Kessler MJ., Berard JD, Schmidtke J, et al. A
562 longitudinal study of age-specific reproductive output and body condition among male
563 rhesus macaques, *Macaca mulatta*. Naturwissenschaften. 2003;90: 309–312.
564 78. Dubuc C, Ruiz-Lambides A, Widdig A. Variance in male lifetime reproductive success and
565 estimation of the degree of polygyny in a primate. Behav Ecol. 2014;25: 878–889.
566 79. Kuester J, Paul A, Arnemann J. Age-related and individual differences of reproductive
567 success in male and female Barbary macaques (*Macaca sylvanus*). Primates. 1995;36: 461–
568 476.
569 80. Bercovitch FB. Social stratification, social strategies, and reproductive success in primates.

570	Ethol Sociobiol. 1991;12: 315–333.

571 81. Strum SC. Reconciling aggression and social manipulation as means of competition: 1.
572 Life'hist0ry perspective. Int J Primatol. 1994;15: 739–765.
573 82. Waitt C, Little AC, Wolfensohn S, Honess P, Brown AP, Buchanan-Smith HM, et al.
574 Evidence from rhesus macaques suggests that male coloration plays a role in female
575 primate mate choice. Proc R Soc Lond B. 2003;270: S144–S146.
576 83. Dubuc C, Allen WL, Maestripieri D, Higham JP. Is male rhesus macaque red color
577 ornamentation attractive to females?. Behav Ecol Sociobiol. 2014;68: 1215–1224.
578 84. Petersdorf M, Dubuc C, Georgiev AV, Winters S, Higham JP. Is male rhesus macaque facial
579 coloration under intrasexual selection?. Behav Ecol. 2017;28: 1472–1481.
580 85. Gazes RP, Schrock AE, Leard CN, Lutz MC. Dominance and social interaction patterns in
581 brown capuchin monkey (*Cebus [Sapajus] apella*) social networks. Am J Primatol.
582 2022;84: e23365.
583 86. Silk JB, Beehner JC, Bergman TJ, Crockford C, Engh AL, Moscovice LR, et al. Female
584 chacma baboons form strong, equitable, and enduring social bonds. Behav Ecol Sociobiol.
585 2010;64: 1733–1747.
586 87. de Waal FBM, Luttrell LM. The similarity principle underlying social bonding among
587 rhesus monkeys. Folia Primatol. 1986;46: 215–234.

| 588 |  |
| --- | --- |
| 589 | **Supporting Information** |
| 590 | S1 File. Information for candidate fathers. |
| 591 | (DOCX) |
| 592 | S2 File. Genotype data for 88 individuals of the B group. |
| 593 | (XLSX) |
| 594 | S3 File. Summary for population genetic parameters. |
| 595 | (DOCX) |
| 596 | S4 File. Results for paternity of offspring in the B group. |
| 597 | (DOCX) |

Fig. 1	[Click here to access/download;Figure;Fig. 1.jpg](https://www2.cloud.editorialmanager.com/pone/download.aspx?id=36744143&guid=36d228df-307e-46ac-89bf-e0fb0cf83f93&scheme=1)[<https://www2.cloud.editorialmanager.com/pone/download.aspx?id=36744143&guid=36d228df-307e-46ac-89bf-e0fb0cf83f93&scheme=1>](https://www2.cloud.editorialmanager.com/pone/download.aspx?id=36744143&guid=36d228df-307e-46ac-89bf-e0fb0cf83f93&scheme=1)

Fig. 2	[Click here to access/download;Figure;Fig. 2.jpg](https://www2.cloud.editorialmanager.com/pone/download.aspx?id=36744144&guid=eac83458-3186-4eb2-977d-f265d8e26b7d&scheme=1)[<https://www2.cloud.editorialmanager.com/pone/download.aspx?id=36744144&guid=eac83458-3186-4eb2-977d-f265d8e26b7d&scheme=1>](https://www2.cloud.editorialmanager.com/pone/download.aspx?id=36744144&guid=eac83458-3186-4eb2-977d-f265d8e26b7d&scheme=1)

Fig. 3	[Click here to access/download;Figure;Fig. 3.JPG](https://www2.cloud.editorialmanager.com/pone/download.aspx?id=36744145&guid=99a14cf1-4d4a-4f4c-9929-ad7570563336&scheme=1)[<https://www2.cloud.editorialmanager.com/pone/download.aspx?id=36744145&guid=99a14cf1-4d4a-4f4c-9929-ad7570563336&scheme=1>](https://www2.cloud.editorialmanager.com/pone/download.aspx?id=36744145&guid=99a14cf1-4d4a-4f4c-9929-ad7570563336&scheme=1)

Table S1

[Click here to access/download](https://www2.cloud.editorialmanager.com/pone/download.aspx?id=36744146&guid=eb962d2d-c576-4795-b244-d5e4b55ceeb6&scheme=1)

[Supporting Information Table S1.docx](https://www2.cloud.editorialmanager.com/pone/download.aspx?id=36744146&guid=eb962d2d-c576-4795-b244-d5e4b55ceeb6&scheme=1)

Table S2

[Click here to access/download](https://www2.cloud.editorialmanager.com/pone/download.aspx?id=36744148&guid=22fdf642-4f44-4b48-9153-40f1ab9f6ce6&scheme=1)

[Supporting Information Table S2.xlsx](https://www2.cloud.editorialmanager.com/pone/download.aspx?id=36744148&guid=22fdf642-4f44-4b48-9153-40f1ab9f6ce6&scheme=1)

Table S3

[Click here to access/download](https://www2.cloud.editorialmanager.com/pone/download.aspx?id=36744150&guid=257d3dc1-0348-45b1-8a0d-c16c90a3d61c&scheme=1)

[Supporting Information Table S3.docx](https://www2.cloud.editorialmanager.com/pone/download.aspx?id=36744150&guid=257d3dc1-0348-45b1-8a0d-c16c90a3d61c&scheme=1)

Table S4

[Click here to access/download](https://www2.cloud.editorialmanager.com/pone/download.aspx?id=36744151&guid=b6b9b4ed-80ab-4b9a-8ef5-fab9635e1178&scheme=1)

[Supporting Information Table S4.docx](https://www2.cloud.editorialmanager.com/pone/download.aspx?id=36744151&guid=b6b9b4ed-80ab-4b9a-8ef5-fab9635e1178&scheme=1)

Response letter

**Response letter**

Reviewer #1: The study “Paternity success for resident and non-resident males and their influences on paternal sibling cohorts in Japanese macaques on Shodoshima Island”, by Ishizuka and collaborators is an interesting report of the reproductive dynamics in Macaca fuscata in Choshikei Monkey Park. The authors identify that a single non-resident male sired almost a third of the individuals born between 2020 and 2022, contradicting what would be expected from social species. This is a very interesting finding, and the manuscript would benefit from the authors bringing the discussion one step further. My comments are written bellow:

- We sincerely thank your review and helpful comments on our manuscript. Based on your comments, we have revised our manuscript significantly. We have responded to each of your comments in detail as follows.

In the “Study subjects” section of the Material and Methods, the authors state that the macaques are “provisioned by park staff (…) and by tourists visiting the park”. Could the feeding have decreased the time the resident macaques spend foraging and permitted them to invest higher energy in reproduction? Also, I would imagine that a higher number of well- fed adult females would mean a higher number of estrous females, lowering the competition pressure among resident and non-resident males. Do the authors think that could interfere with the results obtained?

- As you indicated, the effects of provisioning on their reproduction are possible. So it is important to be careful of interrupting the results.

Many previous studies have been conducted from provisioned populations. Our results were basically consistent with previous results regarding Japanese macaques. Therefore, we considered that our results reflect the general patterns of this species. We thus considered that our data can contribute to better understanding the primate reproductive ecology.

Similarly, the ratio of female to male changed through the years analyzed. How would this impact the results?

- That also can impact the results. Therefore, we included the number of capable females as an explanatory variable in GLMMs. Such effect on paternity success was not significant.

I understand that EGP is calculated through the number of offspring that is sired from non- resident and resident males. But that does not translate directly to reproductive success, which would be calculated using the number of mating attempts by one group of individuals. A better proxy for success would be the number of offspring sired divided by the total number of individuals of each group (residents and non-residents). If there is a higher number of approaching non-resident macaques, them siring 50% of the offspring would mean they have a lower success rate than the resident ones. How many non-resident males were identified, even if they were not sampled?

- The idea that divides the number of sires by the total number of resident or non-resident males may be a good way. However, the total number of non-resident males remains unclear even though most of them were identified. Therefore, we considered that assessing the number of sires for each male (including both resident and non-resident males) should be reasonable.

We identified four non-resident males. We successfully collected samples for three of the four males. We have explained these as below:

L160-161: “We identified four non-resident males and three of them were successfully sampled.”

I feel some information of the group dynamics of Japanese macaques would be useful for the reader to better interpret the results. Do young males usually disperse or stay within the mother’s group after reaching maturity? How related are the adult macaques in the analyzed group? How many years does resident males usually stay in a group and how long it takes for non-resident male to become resident? Does the group change composition usually change between mating and non-mating season? These are some interesting points for the authors to discuss regarding the data obtained.

- As you suggested, we added basic information of Japanese macaques, including group composition, males’ dispersal and tenure, and dominance style. Relatedness among adults was not analyzed in this study. The group composition usually does not change between mating and non-mating seasons. Several non-resident males sporadically appear around the group during mating seasons, and some of them continue to stay in the group after the mating season ends. In such cases, those males are interrupted to immigrate into the group.

L104-110: “They form multi-male and multi-female bisexual groups, in which females stay philopatric and form strong affiliative relationships with matrilineal female kin [44–51]. Males typically emigrate from their natal group when they are approximately four or five years old [52], and continue to transfer between groups throughout life [53]. The tenure of males in a group is approximately three years [54]. Dominance rank is basically linear and stable in both sexes [54– 56], although troop takeovers by non-troop males were reported from the Yakushima population [57].”

The results imply that, in the short term, being a non-resident male would be more advantageous because of the higher reproductive success. Still, group behavior is maintained throughout the Primates clade, including Macaca. Is there a higher survival rate of resident members when compared to non-residents? A longer lifespan may compensate the short-term lower success rate by having a higher total number of offspring and by avoiding putting all reproductive effort on harsh seasons.

- What you indicated is important. However, it is extremely hard to monitor the life history of non-resident males in the wild. We have not found any data that compared the survival rate between resident and non-resident males.

I recommend the authors add to the supplement materials the individual genotypes for the 16 microsatellites analyzed.

- As you suggested, we added the genotype data in Table S2.

Small comments:

- In the abstract, line 41: The phrase “neither higher nor lower” should be changed to “similar”.
- We revised as you suggested.
- In line 67, the sentence “Therefore, the exact number of offspring sired by each non- resident male has been rarely investigated.” is redundant when considering the previous one and should be removed. Same thing happens to the sentence started with “Therefore…” in line 90.
- In line 143, change “although all non-resident males were not sampled” to “although most of the non-resident males were not sampled”.
- We added the information about the number of identified non-resident males here. We have revised this part as follows:

L160-161: “We identified four non-resident males and three of them were successfully sampled.”

Reviewer #2: OVERALL COMMENTS:

I am very excited about the topic and data presented in this study. With some careful revision and a clear theoretical structure, this paper will make a valuable contribution to the literature. My specific comments below are sincerely meant to be constructive and encouraging, though I recognize they are extensive. I wish the authors the utmost best in reconsidering their approach and framework for communicating this study.

- We sincerely thank your review and helpful comments on our manuscript. Based on your comments, we have revised our manuscript significantly. We have responded to each of your comments in detail as follows.

Overall, I urge the authors to:

1. Reframe their study with a detailed, species-specific introduction and a clear theoretical approach (e.g. benefits of dominance, alternative mating strategies, female choice, etc.).
   - As you suggested, we have added the detail information about Japanese macaques (L102- 122). We have also explained the effects of dominance hierarchies and female choice on male reproductive success (L55-64). Please review the revised Introduction.
2. Carefully proof-read the manuscript. There are a number of grammatical errors or careless errors like tables and text disagreeing with each other.
   - We have checked the grammatical or careless errors. We have also outsourced the English correction service as the second round. We apologize for the mistakes.
3. Clarify the methodology.

and statistical methodology (L215-218).

ABSTRACT:

L29-30 I don’t think you need to broadly discuss mammals. I would focus on primates instead.

- As you suggested, we have specifically focused on primates throughout the manuscript. L32 I would change “males” to “individuals” to avoid over-using the word “male” or “males”
- We have used “individuals” as you suggested (L34).

L29-34 I was surprised, given the topic, to see no mention of the role of dominance or alternative mating strategies. See my comments on the introduction for more detail.

- As you suggested, we have explained the effects of dominance and female mate choice on male reproductive success in the Abstract. We have revised as follows:

L31-33: “As resident males achieve different degree of reproductive success with regard to various factors, such as male dominance relationships or female mate choice, the degree of reproductive success for non-resident males may vary between individuals.”

L34-38 You say that you examined paternity for 46 individuals, but later say you could only successfully assign paternity for 34 offspring. So didn’t you really examine paternity for 34 individuals?

- As you suggested, we have revised to state that we examined paternity of 34 offspring (L40).

L38 You assessed paternity success, but you also compared it between individuals – correct? I would highlight this.

- Yes, we compared paternity success between males.

L39-40 “We investigated the percentages of paternal sibling dyads…” I don’t understand what this sentence is trying to say.

L42-43: “We quantitatively assessed the percentages of paternal siblings in the same age cohorts”

L41 “Non-resident males sired neither higher nor lower percentage of offspring” – so doesn’t this mean that they sired an equal percentage of offspring?

- We have revised to state “similar” percentage (L43).

L42 Was it the same resident male who was successful both years? Or two different resident males?

- it was the same NON-resident male (The ID was “NP”).

L44-45 This point about female preference seems to come from nowhere and it isn’t clear how or why this would explain your results especially since you haven’t discussed female choice or mating strategies at all.

- Since we do not have quantitative data on female choice, we stopped discussing the effects of female choice in the Abstract. However, we have discussed the effects of female choice on male reproductive success in the Introduction and Discussion.

INTRODUCTION

1. You use the word “rarely” quite a bit in your manuscript. I would remove some of these or exchange for different words.
   - We have changed “rarely” to different phrases. In the revised manuscript, the “rarely” has been used only once (L30).
2. You spend a lot of time talking about group-living mammals. The phenomenon you are studying is common enough among primate species that I think you should dive right into primate-specific literature.
   - As you suggested, we have specifically focused on “primates” throughout the manuscript.
3. Speaking of primate-specific literature, your introduction is incredibly broad and surface- level. You provide very limited specific theoretical framework, concrete examples,
   - We have added more explanation about the effects of dominance ranks (L56-60) and female mate choice (L60-64) on male reproductive success.
4. Japanese macaques are you focal species, but the only information you provide is “They form female-philopatric and multimale/multifemale groups”. This is not nearly enough species-specific information.
   - We have added more information about Japanese macaques, such as, group composition, female nepotisms, male dispersal, dominance style, breeding seasonality, and female mate choice. We have revised as follows:

L102-122: “To investigate the paternity success for non-resident males and their contributions to the production of kin-dyads within groups, the Japanese macaque *Macaca fuscata* is an interesting species. They form multi-male and multi-female bisexual groups, in which females stay philopatric and form strong affiliative relationships with matrilineal female kin [44–51]. Males typically emigrate from their natal group when they are approximately four or five years old [52], and continue to transfer between groups throughout life [53]. The tenure of males in a group is approximately three years [54]. Dominance rank is basically linear and stable in both sexes [54– 56], although troop takeovers by non-troop males were reported from the Yakushima population [57]. Noteworthily, non-resident males often breed with females of the group, as the percentages of EGP are relatively high among primates [28,31]. This is partially because they have breeding seasonality [58,59], and novel and unfamiliar males attain high reproductive success because they are strongly selected by females for mating partners when females are likely to conceive [19,21,60,61]. Further, the exact number of siring for each non-resident male in the field can also be assessed. During the mating season, several non-resident males sporadically appear around the group and copulate with the group’s females [24,62]. Considering that previous field research succeeded in collecting behavioral data and genetic samples from non-resident males [19,24,63], it is possible to expand on it further to examine the paternity success for non-resident males at the individual level. Moreover, the percentage of paternity by the highest-ranking male is usually low [19,24,64], although one study reported a relatively high percentage [65]. This suggests that the percentages of paternal sibling dyads produced by non-resident males can be assessed quantitatively.”

5. Please revise your introduction to be more concise. The second paragraph particularly is rather repetitive.
   - We have revised the Introduction significantly. To avoid the redundancy of the paragraph, we have deleted several unnecessary words or sentences. The total number of words in the paragraph has been changed from 305 to 219.
6. I highly recommend that the authors consider a new framework for this paper that focuses on dominance and alternative mating strategies. This would give you a clearer platform from which to launch your investigation of paternity success and the prevalence of EGP. There are some really great articles on this topic in primates and Japanese macaques particularly. I would consider adding any of the following:
a. Bernstein, I. (1976). Dominance, aggression and reproduction in primate societies. Journal of Theoretical Biology, 60(2), 459–472.
b. Bernstein, I. (1978). Primate dominance hierarchies. La Recherche, 9(91), 663–668
c. Chapais, B. (1983). Reproductive activity in relation to male dominance and the likelihood of ovulation in rhesus monkeys. Behavioral Ecology and Sociobiology, 12, 215–228
d. de Ruiter, J. (1993). Male dominance rank and reproductive success in primate groups. Primates, 34(4), 513–523.
e. Eaton, G. G. (1974). Male dominance and aggression in Japanese macaque reproduction. Advances in Behavioral Biology, 11, 287–297. https:// doi.org/10.1007/978-1-4684-3069- 1_13.
f. Eaton, G. G. (1976). The social order of Japanese macaques. Scientific American, 235(4), 96–107.
g. Furuichi, T. (1983). Interindividual distance and influence of dominance on feeding in a natural Japanese macaque troop. Primates, 24(4), 445–455
h. Hayawaka, S., & Soltis, J. (2011). Troop takeover and reproductive success of wild male Japanese macaques on Yakushima Island (Macaca fuscata yakui). International Journal of Zoology, 2011, 1–8.
i. Kutsukake, N., & Nunn, C. L. (2006). Comparative tests of reproductive skew in male primates: The roles of demographic factors and incomplete control. Behavioral Ecology and Sociobiology, 60(5), 695–706. https://doi.org/10.1007/s00265-006-0213-1
j. Kutsukake, N., & Nunn, C. L. (2006). Comparative tests of reproductive skew in male primates: The roles of demographic factors and incomplete control. Behavioral Ecology and Sociobiology, 60(5), 695–706. https://doi.org/10.1007/s00265-006-0213-1
k. Perloe, S. I. (1992). Male mating competition, female choice and dominance in a free‐ ranging group of Japanese macaques. Primates, 33(3), 289–304
l. Takahata, Y., Huffman, M. A., Suzuki, S., Koyama, N., Yamagiwa, J., & Yamigawa, J. (1999). Why dominants do not consistently attain high mating and reproductive success: A review of

longitudinal Japanese macaque studies. Primates, 40, 143–158. https://doi.org/10.1007/ BF02557707

- Thank you for sharing many important previous researches. We have cited most of these studies (Berstein 1976; De Ruiter 1993; Eaton 1976; Furuichi 1983; Hayakawa & Soltis 2011; Kutsukake & Nunn 2009; Perloe 1992; Takahata et al. 1999) and revised our manuscript significantly.

METHODS

1. It feels like there is a lot of information missing here.
   - We have added more information and revised the Methods significantly.
2. L124 says that the table is for 2019-2020, but the table title says 2019-2021. This is an example of the much-needed proof-reading.
   - We apologize for the careless error. We have revised here.
3. Please describe the “daily monitoring” in depth
   - We have explained the “daily monitoring” in detail as follow:

L135-139: “In April 2018, we started monitoring the presence of identified individuals in the monkey park on a daily basis (five or six days per week), although their presence has not been quantitatively recorded. In February 2019, we successfully completed individual identification of the group, and started systematically recording the daily presence of the members of the group.”

3. L128-129 casually mentions capturing of individuals for wildlife management? This needs to be explained in detail since it could be a significant limitation or influencing element of your study
   - From 2019 to 2020, many monkeys disappeared one by one. At the time, we did never observed corpses of the monkeys, so did not know reasons for the disappearance. After a couple of years, we found the governmental documents reporting that the monkeys were captured during the period in the town that includes our study site. Therefore, we suspect that the disappearance was due to the captures by the government. However, there is no evidence confirming that the animals were actually captured by the government.

As you pointed out, the effect of the disappearance on the results can be possible. However, it still remains unclear whether the disappearance was a natural catastrophic event, or was unnaturally caused by humans. Therefore, we considered that describing just the fact of disappearance and its possible reasons should be appropriate in the manuscript.

4. Looking at Table 1, only 41/81 group members are accounted for. Are the other 41 juveniles and infants? Please expand this table.
   - Yes, we have indicated them in Table 1.
5. Three non-resident males were identified, but how many resident males? Of those, how many were dominant or subordinate? We need a lot more details here.
   - We have clarified the number of resident males, including dominant and subordinate males, between 2019 and 2021 as follows:

L171-172: “Two to three dominant, eight to ten subordinate, and two identified non-resident males were present between 2019 and 2021, respectively.”

6. Japanese macaques form despotic linear hierarchies. Why was no effort made to look at the hierarchy using a David’s Score or an Elo Rating or some other measure?
   - As you pointed out, we agree the importance of dominance hierarchies in this study. However, it was difficult to collect the dominance-related behaviors of males throughout the five years.

The observation of the study subjects was mainly conducted by YK, who is a park staff of the monkey park and is coauthored in this submission. She could not collect systematic behavioral data, such as antagonistic interactions or signals of submission, because of her own jobs as the park staff. Therefore, there is no data to construct the dominance hierarchies based on the David’s score or Elo rating.

8. Your GLMM – was this using a binomial distribution? What was your link function? Overall, this needs to be substantially expanded with a lot more specific information about your processes.
   - We have explained the error distribution and link function. To clarify the processes of statistical analyses, we have added more explanation about the response variables, and model selection. We have revised as follows:

L213-218: “To analyze the likelihood of siring by each male, we constructed generalized linear mixed models (GLMMs) with binomial distribution and logit link function. We included the percentage of sires for each male in each year as a response variable. Since the total number of sampled males was 10, 10, 12, 12, and 13 in 2017, 2018, 2019, 2020, and 2021, respectively, the total number of data points was 57. The response variable ranged from 0 to 0.40, as the most successful sire’s share in a year was 40% (see the Results).”

I don’t feel I can assess the RESULTS and DISCUSSION based on my concerns and confusion regarding the INTRODUCTION and the METHODS.

- We have revised the Introduction and Methods significantly. We hope that you can assess the Results and Discussion of this study. We are glad if you provide comments on our manuscript.
